# Initial assessment of all-season Arctic sea ice thickness from ICESat-2

**DOI:** 10.1017/jog.2025.10119

**Published:** 2025-12-26

**Authors:** Alek Petty, Alex Cabaj, Jack C. Landy

**Affiliations:** 1 Earth System Science Interdisciplinary Center, University of Maryland, College Park, MD, USA; 2 Climate Research Division, Environment and Climate Change Canada, Toronto, ON, Canada; 3 Earth Observation Group, Institute of Physics and Technology, UiT The Arctic University of Norway, Tromsø, Norway

**Keywords:** laser altimetry, sea ice, sea-ice growth and decay

## Abstract

We present an initial assessment of all-season Arctic sea ice thickness estimates from ICESat-2 by combining freeboard retrievals with all-season SnowModel-LG snow loading. ICESat-2 captures the key regional and seasonal patterns of Arctic sea ice variability and shows good agreement with CryoSat-2 all-season estimates, including regional patterns of inter-annual variability in summer ice thickness. ICESat-2 shows consistently thicker ice compared to CryoSat-2 across the western coastal Arctic, while CryoSat-2 shows some periods of thicker ice across the Central Arctic, largely consistent with winter thickness biases. Validation against upward-looking sonar moorings, IceBird-2019 airborne observations and MOSAiC buoy data highlights generally strong performance across a range of conditions, although seasonal biases linked to snow loading, freeboard differences and ice density assumptions persist. The SnowModel-LG and NESOSIM snow accumulation models perform well across the validation datasets, but do not consistently add skill beyond the modified Warren climatology. Experimental ICESat-2/CryoSat-2 dual altimetry winter snow depths show strong performance relative to existing products and future work should extend these into summer for further assessments. Overall, our analysis supports the viability of an all-season ICESat-2-derived thickness record.

## 1. Introduction

Monitoring Arctic sea ice from space is crucial for understanding our rapidly changing polar regions. Satellite observations provide arguably the best means to consistently track variability and trends in sea ice conditions due to their combination of reliability, basin-scale coverage and multi-year mission duration (Meier and others, 2014; Kwok, 2018; Stroeve and Notz, 2018). Satellite-derived sea ice observations are routinely used to assess and calibrate climate models (Notz and Stroeve, 2016; Massonnet and others, 2018), and understand the impacts of sea ice loss on global weather patterns (Cohen and others, 2014), ocean circulation (Polyakov and others, 2023) and Arctic ecosystems (Post and others, 2013).

NASA’s ICESat-2 mission has significantly enhanced our ability to routinely monitor sea ice conditions at high resolution across the polar oceans (Markus and others, 2017). The data obtained from ICESat-2’s Advanced Topographic Laser Altimeter System (ATLAS) over sea ice include estimates of height and total freeboard, the vertical extension of sea ice and its overlying snow cover above local sea level. The official ICESat-2 sea ice data products include along-track height (ATL07) and total freeboard (ATL10) for each of the six laser beams, as well as monthly gridded estimates of total freeboard (ATL20) and sea surface height anomalies (ATL21). These data are routinely updated and made available through the National Snow and Ice Data Center (NSIDC). ICESat-2’s year-round data acquisition has provided a continuous record to monitor total freeboard variability since its launch in fall 2018 (Kwok and others, 2019b; Petty and others, 2023a, 2023b). The along-track sea ice height and total freeboards have demonstrated high precision and accuracy compared to coincident airborne data collected in spring 2019 (Kwok and others, 2019a), with further validation across seasons and regions still ongoing. ICESat-2 extends the laser altimetry record of sea ice obtained by the original ICESat mission (2003–09) (Kwok and Cunningham, 2008; Petty and others, 2020) and provides a crucial complement to radar altimetry data obtained by the European Space Agency’s (ESA) CryoSat-2 mission, which has been operational since its launch in 2010 (Ricker and others, 2017; Kwok, 2018; Tilling and others, 2018; Landy and others, 2022).

Estimates of winter Arctic sea ice thickness have been produced from ICESat-2 total freeboards using the traditional approach of assuming hydrostatic equilibrium and prescribing snow loading and ice density (Petty and others, 2023c). Both along-track (IS2SITDAT4 Version 1, Petty and others (2022)) and monthly gridded (IS2SITMOGR4 currently at Version 3, Petty and others (2023c)) datasets are made available through the NSIDC, with the monthly gridded dataset updated annually. These winter Arctic sea ice thickness estimates have primarily utilized snow loading estimates from the NASA Eulerian Snow on Sea Ice Model (NESOSIM) (Petty and others, 2018) to-date. Updates to NESOSIM (now at Version 1.1) and, more significantly, updates to the underlying ICESat-2 freeboards (Release 006 at the time of writing) have improved agreement with various CryoSat-2 winter Arctic sea ice thickness estimates (Kwok and others, 2021; Petty and others, 2023b). The thickness estimates are currently produced for the Arctic winter months only (September through April) due to the unavailability of NESOSIM snow loading in summer (no inclusion of summer melt processes to-date) and concerns around freeboard data reliability in summer. The ICESat-2 sea ice surface finding algorithm is not currently able to discriminate melt ponds from open water leads or sea ice; however, various filters are in place that are expected to mitigate this potential impact on the derived sea surface height and total freeboard estimates (Tilling and others, 2020; Kwok and others, 2020a, 2025). A summer airborne ICESat-2 cal/val campaign was undertaken in 2022, which included data collected by both the Land, Vegetation and Ice Sensor (LVIS) and Leica Chiroptera-4x (CHIR) lidar systems (Saylam and others, 2025) as well as coincident georeferenced imagery (Blair and others, 2023). Preliminary results indicate encouraging performance of the summer ICESat-2 height retrievals (Saylam and others, 2025), but no comprehensive evaluation of summer total freeboard has been undertaken to-date.

In contrast, recent advances in data processing techniques have enabled new all-season ice freeboard estimates from ESA’s CryoSat-2 (Dawson and others, 2022), which have been combined with all-season Arctic snow loading estimates from the SnowModel-LG Lagrangian accumulation model (Liston and others, 2020) to produce all-season estimates of Arctic sea ice thickness (Landy and others, 2022). These data have provided crucial new insights into seasonal and regional thickness anomalies and forecast skill relevant to various Arctic stakeholders. In addition, new methods to derive snow depth and sea ice thickness concurrently by fusing altimetry data across missions/sensors, e.g. CryoSat-2’s radar-derived ice freeboards and ICESat-2’s laser-derived total freeboard, and leveraging their contrasting profiling assumptions is an active area of research focus and promise (Kwok and others, 2020b; Kacimi and Kwok, 2022; Fredensborg Hansen and others, 2024; Landy and others, 2024; Carret and others, 2025).

In this study, we seek to extend the winter (September to April) ICESat-2 derived sea ice thickness estimates into summer and provide a first assessment of these data relative to the existing CryoSat-2 all-season estimates. We utilize the all-season snow loading estimates from SnowModel-LG to ensure consistency with the all-season CryoSat-2 product (Landy and others, 2022) and to provide clearer insights into the impact of altimetry differences on resultant sea ice thickness. Although summer freeboard validation is lacking, we instead carry out indirect validation by comparing the new all-season ICESat-2-derived thickness estimates with a variety of independent datasets: (i) under-ice upward-looking sonar (ULS) mooring ice draft data from the Beaufort Gyre Exploration Project (BGEP) (Krishfield and others, 2014), (ii) multisensor airborne data collected during the 2019 AWI IceBird campaign (Jutila and others, 2024), (iii) snow and ice thickness buoy observations collected during the MOSAiC expedition (Lei and others, 2022). The BGEP dataset has a strong legacy of supporting altimetry-derived Arctic sea ice thickness validation (Landy and others, 2022; Petty and others, 2023b) and provides consistent data across multiple years; however, the moorings are limited to a fixed Beaufort Sea region. The MOSAiC and IceBird datasets provide crucial additional data across other regions of the Arctic, albeit limited in time. We include the CryoSat-2 all-season dataset, an experimental CryoSat-2/ICESat-2 derived winter Arctic dual altimetry fusion snow depth dataset (Landy and others, 2024) and additional input assumptions in our analysis and assessments to provide context for our all-season ICESat-2 thickness assessments. The overall objectives of this study include:

1. Incorporate SnowModel-LG all-season snow depth and density estimates into ICESat-2 thickness retrievals, providing an initial estimate of all-season ICESat-2 Arctic sea ice thickness.
2. Compare the new all-season ICESat-2 thickness results with the current state-of-the-art CryoSat-2 derived all-season thickness estimates.
3. Assess the new summer and existing winter ICESat-2 and all-season CryoSat-2 thickness estimates with independent Arctic snow depth and thickness datasets, highlighting the seasonal and regional biases across datasets.
4. Provide new insights into the performance of altimetry-derived Arctic sea ice thickness estimates and help motivate future development efforts.

All of the analysis presented in this study is provided publicly in a series of online interactive notebooks (https://www.icesat-2-sea-ice-state.info), extending the analysis that supported our winter thickness assessments (Petty and others, 2023b). The notebooks provide transparency and guidance on how to implement similar comparisons, as well as enabling interested users to implement different filtering or masking options as desired.

## 2. Data and methods

### 2.1. ICESat-2

#### 2.1.1. IS2SITMOGR4 winter Arctic monthly gridded sea ice thickness

We use monthly gridded winter Arctic sea ice thickness estimates from ICESat-2 (IS2SITMOGR4) disseminated through the National Snow and Ice Data Center (NSIDC) https://nsidc.org/data/is2sitmogr4). This monthly gridded dataset is derived from along-track ICESat-2 total freeboard measurements (ATL10) and the hydrostatic equilibrium assumption using snow loading estimates from the NASA Eulerian Snow On Sea Ice Model (NESOSIM, Version 1.1) (Petty and others, 2018, 2023b), a constant bulk ice density of 916 kg m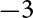 and seawater density of 1024 kg m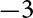. Sea ice thickness is first calculated along-track for the three strong beams of ICESat-2, using an empirically-derived snow redistribution scheme described in Petty and others (2020). The underlying ATL10 freeboards are limited to regions of ice concentration 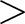
50% based on daily passive microwave observations. The along-track thickness data from the three strong beams are binned monthly to a 25 km x 25 km North Polar Stereographic grid across the entire Arctic.

The Version 3 IS2SITMOGR4 data were described in Petty and others (2023b) and utilized Release 006 ATL10 total freeboard data. The thickness dataset included estimates of winter Arctic sea ice thickness derived with different input assumptions, which we utilize and extend in this study for basic insights into the impact and sensitivity of our comparisons to the choice of input assumptions.

(i) SMLG: SnowModel-LG snow depth and density (Liston and others, 2020) and the resultant winter sea ice thickness were included in the IS2SITMOGR4 v3 dataset. SMLG integrates reanalysis-based atmospheric forcing from either the ERA5 or MERRA-2 reanalysis with a Lagrangian snow accumulation scheme, and a 3D snow transport model accounting for blowing snow redistribution (deposition and erosion) and sublimation mass loss—processes that are not explicitly represented in NESOSIM. SMLG also includes parameterized snow melt and metamorphosis processes by accounting for energy transport within the snowpack, which is likewise not represented in NESOSIM and thus provides a clear limitation on its utility in producing spring/summer snow loading (future NESOSIM developments are working to introduce this as highlighted in the later Discussion section). A detailed description of SMLG is available in the Appendix of Liston and others (2020). Only ERA5-forced SM-LG data were provided in IS2SITMOGR4 v3 to be consistent with the ERA5-forced NESOSIM. For this study and the new winter thickness dataset, we in addition utilize SMLG snow loading based on MERRA-2 forcing to be consistent with the snow loading used by the CryoSat-2 all-season product described below and to explore the sensitivity to differences in snow loading. It is worth noting that snowfall in SMLG is derived from (water-equivalent) reanalysis precipitation (using a phase fraction parameterization as described in Liston and others (2020)), whereas NESOSIM uses the reanalysis snowfall rate directly. SMLG also applies corrections to precipitation including clipping and redistributing ‘drizzle’ values (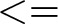 1 mm/day water equivalent) to discrete precipitation events and bias correction of the precipitation to improve agreement with snow depth estimates from NASA’s Operation IceBridge mission. This makes it challenging to directly assess the relative role of reanalysis forcing differences on the resultant SMLG snow depths. Note that the SMLG data are available publicly through July 2021.

(ii) MW99: the Warren snow depth/density climatology (Warren and others, 1999), modified such that snow depth is halved over first-year ice (FYI). Although this climatology was compiled from observations collected several decades ago, it provides a useful additional comparison to the accumulation models, and in-situ surveys continue to suggest it can provide realistic snow distributions over mature FYI and multiyear ice (MYI) (Haas and others, 2017). We use the Inner Arctic monthly mean values of this climatology, as in Tilling and others (2018).

(iii) J22: the variable empirical bulk ice density parameterization of Jutila and others (2022), a function of the along-track ice freeboard (total freeboard minus snow depth). This was produced using multi-sensor airborne data collected over regions of the western Arctic in April only, so its relevance to other regions and seasons is unknown.

These additional input assumptions and resultant sea ice thicknesses form the basis of a new Version 4 release of the IS2SITMOGR4 winter thickness dataset, available initially from November 2018 to April 2025, covering the Arctic Ocean between September and April only (Petty et al., 2025).

#### 2.1.2. IS2SITMOGR4S summer Arctic monthly gridded sea ice thickness

To extend ICESat-2 ice thickness retrievals across the summer months of May through August, we derive additional monthly gridded sea ice thickness estimates (denoted as IS2SITMOGR4S) using both ERA5 and MERRA-2 forced SMLG snow depth and density estimates (Liston and others, 2020) as in the winter data above. We use the exact same processing chain as in IS2SITMOGR4 to convert along-track ATL10 total freeboards to thickness, using a constant bulk ice density of 916 kg m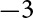 and seawater density of 1024 kg m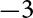 and the empirical snow redistribution scheme. Again, the SMLG data are available publicly through July 2021, which provides a limit on our resultant thickness dataset to the November 2019 to July 2021 time-period. IS2SITMOGR4S browse images for June 2020 using both the ERA5 and MERRA-2 forced SM-LG snow loading are included in the Supplementary Material (Figs. S1 and S2, respectively).

As noted in the introduction, the ICESat-2 sea ice surface finding algorithm is not currently able to discriminate melt ponds (or wet snow) from open water leads or sea ice. However, various filters are in place that are expected to mitigate this potential impact on the derived sea surface height and freeboard, as described in the ICESat-2 sea ice Algorithm Theoretical Basis Document (ATBD, Kwok and others (2025) and summarized in Tilling and others (2020) and Kwok and others (2020a). These include: i) the use of a dual Gaussian fitting procedure that preferentially selects the upper surface as the provided single segment height when two distinct distributions are detected (thus hopefully mitigating the inclusion of any melt pond bottom returns), ii) a requirement for only relatively ‘smooth’ height segment surfaces to be used for sea surface determination (limiting the potential inclusion of mixed surfaces, e.g. sea ice and melt ponds), iii) a height filter applied to the sea surface finding routine that selects only the lowest heights from the height distribution in the given 10 km freeboard section. Our aim with this study is to use the ATL10 data as is and to provide a further indirect validation of the derived summer freeboard, as in Kwok and others (2020a), but through our production and assessment of the derived summer/all-season thickness estimates.

Between June 25th and July 9th, 2019, ICESat-2 entered safehold mode due to a spacecraft anomaly, with no data collected during this period. After leaving safemode, the spacecraft was collecting data nominally, but had issues related to pointing, degrading the radiometry and lead finding performance. No ATL10 freeboard data were generated until nominal pointing resumed around July 25th, 2019. This is the longest period of missing ATL10 data in the ICESat-2 sea ice data record and is summarized in the ATL10 NSIDC Known Issues document (https://nsidc.org/sites/default/files/documents/technical-reference/icesat2_atl07_10_known_issues_v007_0.pdf). Considering the significant missing data, July 2019 was not processed or included in this study. As June 2019 only included six days of missing data, we include that month in our analysis but note the slight skew of representative date toward the start of the month, however overall coverage and segment counts were similar to other months. The Version 1 IS2SITMOGR4S dataset is availble on Zenodo (Petty, 2025).

In addition, we use monthly mean ice passive microwave-derived ice concentration from the NOAA/NSIDC Climate Data Record (CDR), version 4 (Meier and others, 2021), provided in both the IS2SITMOGR4 and IS2SITMOGR4S datasets.

For analysis of both the IS2SITMOGR4 and IS2SITMOGR4S data, we use the provided interpolated/Gaussian smoothed variables as described in Petty and others (2023b), developed to reduce aliasing from uneven monthly sampling and to fill in gaps across small regions of missing data, including the 88 degrees North pole hole. For the interpolated/Gaussian smoothed variables not provided in either product, we apply the same interpolation processing in this analysis for consistency. A 50 % monthly sea ice concentration masking is still applied, as in the underlying freeboard data.

### 2.2. CryoSat-2 all-season ice thickness

We use the University of Bristol (UBRIS) CryoSat-2 all-season sea ice thickness product, Version 1.0 (Landy and others, 2022) available from https://data.bas.ac.uk/full-record.php?id=GB/NERC/BAS/PDC/01613. This dataset provides biweekly gridded Arctic sea ice thickness estimates from October 2010 to July 2021. The period of overlap, November 2018 to July 2021, permits multiple years of comparison with our ICESat-2 derived thickness estimates. Key differences between the CryoSat-2 and ICESat-2 thickness datasets include: i) The UBRIS CryoSat-2 data have been generated with ice-age-dependent ice densities of 917 kg m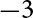 (FYI) and 881 kg m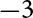 (MYI). The NSIDC weekly 12.5-km sea ice age product V4 (https://nsidc.org/data/nsidc-0611)) was used to differentiate between zones of FYI and MYI. The differences in density for MYI between UBRIS and IS2SITMOGR4S could produce an offset in derived sea ice thickness. ii) The UBRIS CryoSat-2 data are posted biweekly, which we resample here to monthly to be consistent with the monthly ICESat-2 data. iii) The UBRIS CryoSat-2 data are provided at an 80 km spatial resolution on an EASE2.0 grid. We regrid/downsample these to the 25 km Polar Stereographic grid of IS2SITMOGR4 to enable spatial comparison mapping, noting that the data still represents coarser grid-scales.

For the time series analysis we mask all ICESat-2 and CryoSat-2 data outside of an Arctic Ocean domain (includes the Central Arctic, Beaufort Sea, Chukchi Sea, E. Siberian Sea, Kara Sea) to avoid regions that are generally considered more uncertain (wave contamination, snow/flooding, etc.) and focus on the region where our input assumptions and validation data are more representative. The peripheral/masked regions are predominantly sea ice free in late-summer so this masking is more relevant for our winter analysis. In addition, when indicated we utilize a common masking approach, as in Petty and others (2023b), to ensure we only analyze grid-cells where both the ICESat-2 and CryoSat-2 thicknesses are valid for the given month to mitigate against sampling differences between the products. Finally, in some of our spatial comparisons (as indicated) we additionally utilize perennial masking where we only show grid-cells that provide consistent data across all summer months in both ICESat-2 and CryoSat-2 to further avoid sampling biases.

### 2.3. ICESat-2/CryoSat-2 winter Arctic dual altimetry fusion snow depths

We use preliminary winter Arctic snow depth estimates produced through the fusion of ICESat-2 total freeboards and CryoSat-2 ice freeboards, described in Landy and others (2024), with the data provided on Zenodo (https://zenodo.org/records/13774843). Validating these new multi-sensor data are not a primary focus of this study, but we incorporate these data here for added context regarding potential biases in our model-based snow depths and their potential role in driving differences between ICESat-2 and CryoSat-2 derived thicknesses. These data were generated using rel004 ICESat-2 monthly gridded ATL20 freeboards which use the same rel006 along-track ATL10 freeboards as in IS2SITMOGR4 v3 and IS2SITMOGR4S v1.

### 2.4. BGEP ULS ice drafts

**Figure 1.**
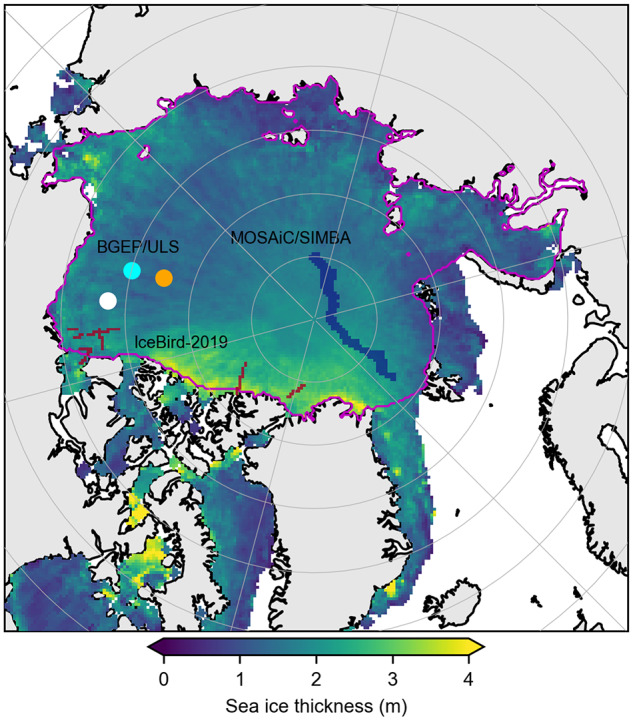
Summer mean (May to August, 2019 to 2021) ICESat-2 sea ice thickness based on MERRA-2 forced SnowModel-LG snow loading (IS2/SMLG-M2), using the interpolated/smoothed data and filled in pole-hole. The solid circles show the location of the BGEP ULS moorings A (cyan), B (orange) and D (mooring C is missing for this period). The dark red lines show the grid-cells covered by the IceBird-2019 data. The dark blue line shows the grid-cells profiled by the MOSAiC/SIMBA buoys. The magenta contour shows the Arctic Ocean domain we use in our time-series analysis (Fig. 5).

The Beaufort Gyre Exploration Project (BGEP) provides upward-looking sonar (ULS) ice draft estimates from three moorings (A, B and D, see Fig. 1) deployed across the Beaufort Sea (Krishfield and others, 2014). The data have been publicly available since the launch of ICESat-2 through August/September 2023 (https://www2.whoi.edu/site/beaufortgyre/data/mooring-data/). Ice draft typically composes a more significant fraction of the total ice thickness compared to the freeboard measured by satellites, depending on the relative densities of ice, seawater and the overlying snow loading, making it a desirable validation metric. Individual ULS draft measurements are assumed to have a constant uncertainty of 10 cm (Krishfield and others, 2014). To compare with the satellite products, we first resample the provided ULS data to daily then monthly means. We then follow the approach of Petty and others (2023b) and take the mean of all valid ICESat-2 grid-cells within a certain radius of a given mooring, and compare this to the monthly mean ULS draft estimate. We use an averaging radius of 100 km in this study, as a compromise between the 50 km used in Petty and others (2023b) and the 150 km used in Landy and others (2022). Petty and others (2023b) explored the impact of averaging radius on BGEP/ULS comparisons and showed only minimal differences between 50 km and 150 km radii.

We calculate ice draft from the ICESat-2 data by simply adding the relevant snow depth to the gridded ice thickness and subtracting the gridded total freeboard. The UBRIS CryoSat-2/SMLG dataset does not include ice freeboard, so we instead estimate this using the hydrostatic equilibrium equation and the provided variables of bulk ice density, snow depth and density and seawater density.

### 2.5. MOSAiC buoy snow depths and ice thickness

The Multidisciplinary Drifting Observatory for the Study of Arctic Climate (MOSAiC) expedition (2019–20) provided high-quality in-situ observations of Arctic sea ice (Nicolaus and others, 2022). We utilize snow depth and sea ice thickness measurements obtained from 19 snow and ice mass balance array (SIMBA) buoys deployed during the MOSAiC expedition (Lei and others, 2022) as independent validation data. SIMBA buoys are thermistor-string-type buoys from which air-snow, snow-ice and ice-water interfaces may be detected, producing measurements of snow and sea ice thickness. The buoys were initially deployed over unponded level ice, and 10 of the buoys collected data for the entire sea ice growth season, providing overall observational coverage from October 2019 to July 2020 (Lei and others, 2022). The buoy tracks drifted from the Eastern Arctic (in October 2019) toward the Fram Strait (spring 2020). Absolute ice thickness and snow depth measurements from individual buoys are not necessarily representative of the average conditions of ice floes in the surrounding area; however, here we are able to benefit from the deployment of a dense network of buoys, which should improve the representation.

The buoy observations are highly localized in nature, so we bin them at daily timescales to a 100 km 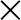 100 km North Polar Stereographic grid. The daily-aggregated data are resampled to monthly gridded means to compare with the monthly gridded satellite estimates. We coarsen the ICESat-2/CryoSat-2 data to the same 100-km resolution before undertaking our comparisons (we require at least four valid 25 km grid points for the 100 km coarsened mean to be used). Higher resolution (25 km) gridded versions of the comparisons between buoys and satellite observations are included in the Supplementary Material as noted (generally showing worse statistical comparisons).

### 2.6. AWI IceBird-2019 snow depths and ice thickness

The Alfred Wegener Institute (AWI) IceBird campaign provides airborne measurements of sea ice thickness and snow depth using combined radar, lidar and (electromagnetic) EM-bird data fusion. We utilize sea ice thickness and snow depth estimates obtained from 5 single-day surveys in April 2019 (April 2, 4, 7, 8 and 10) across the western Arctic Ocean/Beaufort Sea, spanning both first-year and multiyear ice regimes (Jutila and others, 2024). We calculate sea ice thickness from the difference between the sea-ice-plus-snow thickness from electromagnetic induction sensor measurements and coincident snow depth measurements from snow radar data. We exclude measurements where data quality flags indicate negative freeboard, a low number of snow depth estimates within the EM sensor footprint, and implausibly low total thickness values (less than thickness uncertainty, snow depth or snow freeboard). We bin these data daily to the coarser 100 km 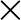 100 km North Polar Stereographic grid as discussed in the previous section, before generating a single April 2019 monthly gridded mean dataset. The higher resolution (25 km) gridded versions are again included in the Supplementary Material as noted.

## 3. Results

### 3.1. All-season sea ice thickness estimates

**Figure 2.**
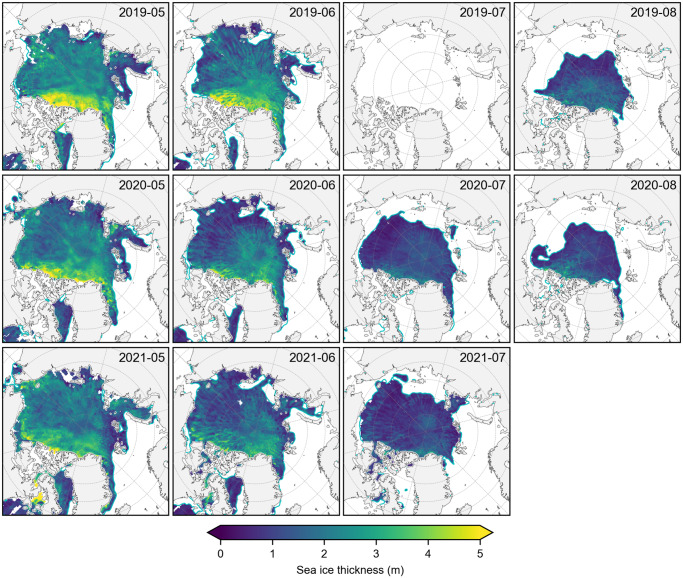
Monthly mean summer (May to August, 2019 to 2021) ICESat-2 Arctic sea ice thickness estimates with MERRA-2 forced SnowModel-LG snow loading, using the interpolated/smoothed gridded data. The cyan contour indicates the 50 % sea ice concentration from the monthly NSIDC CDR v4 dataset.

Figure 2 shows the new summer (May to August) monthly mean Arctic sea ice thickness estimates derived from ICESat-2 total freeboards (rel006 ATL10) and SnowModel-LG-MERRA2 snow loading (IS2/SMLG-M2) for 2019, 2020 and 2021 using the interpolated/smoothed data on the 25 km x 25 km North Polar Stereographic grid. The IS2/SMLG-M2 summer thicknesses show regional distributions largely consistent with the winter freeboards and thickness, including thicker ice along the western Arctic coastline, but also localized regions of thicker ice within the eastern Arctic (Petty and others, 2023b). The expected seasonal decline is clearly evident across all three summers and spatial coverage is consistent across months. The interpolated/smoothed data still show evidence of aliasing due to uneven monthly sampling, especially in June when the sea ice conditions are changing most rapidly within the month. We highlight the need for enhanced gridding/interpolation in the discussion.

**Figure 3.**
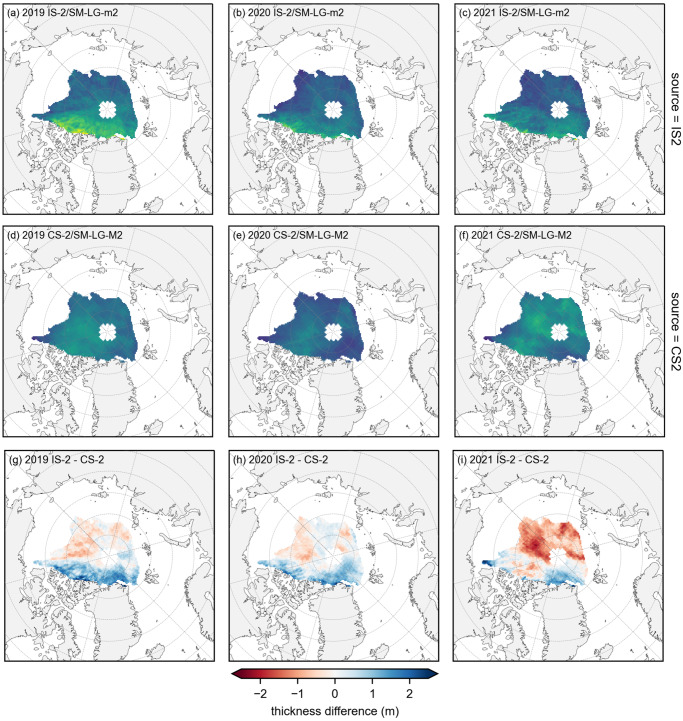
Summer mean (May to August) monthly gridded sea ice thickness from (top row) ICESat-2 and MERRA-2 forced SnowModel-LG (IS2/SMLG-M2), (middle row) CryoSat-2 and SMLG-M2 (CS2/SMLG-M2) and (bottom row) differences between ICESat-2 and CryoSat-2 for 2019 (left), 2020 (middle) and 2021 (right). The monthly data only include grid-cells where both datasets show consistent monthly data across both datasets (a perennial common mask) before averaging. The 2019 means do not include July (missing ICESat-2 freeboards), while 2021 means do not include August (no available SMLG data).

Figure 3 shows spatial difference maps between the IS2/SMLG-M2 summer mean thickness estimates and the summer mean CryoSat-2/SMLG-M2 (CS2/SMLG-M2, UBRIS) data. In these comparisons, we use the perennial/common data masking introduced in Section 2.2 to only show grid-cells that provide consistent monthly coverage across both products to avoid monthly sampling biases contaminating the seasonal averaging (IS2 applies a stricter 50 % sea ice concentration filter and also experiences more data drop-out in general). The use of seasonal means also reduces the aliasing issues discussed in the previous monthly analysis, with the monthly CS2/SMLG-M2 thickness and monthly difference maps provided in the Supplementary Material (Figs. S3 and S4). Overall, there is reasonable agreement between the IS2 and CS2-derived thickness estimates in terms of basin mean thickness but with significant regional differences of up to 1–2 m. IS2/SMLG-M2 shows consistently thicker ice compared to CS2/SMLG-M2 in all three summers across the western central Arctic, especially along the Greenland and Canadian Arctic coastline. However, IS2 also shows some regions of thinner ice compared to CS2 further to the North/East of the Arctic, especially in 2021. The monthly difference maps (Supplementary Material, Fig. S4) also show significant regions in May/June where IS2 is 1–2 m thinner than CS2 across the southern Beaufort/Chukchi seas, and up to 2 m thicker than CS2 in the northern Kara Sea region. While not the main focus of this study, we also produced winter (January through April) IS2 to CS2 thickness differences, which show similar regional thickness biases to summer (Supplementary Material, Fig. S5). Similar regional winter IS2/CS2 thickness biases were also present in comparisons with other CS2 derived thickness products, as featured in the interactive analysis (https://www.icesat-2-sea-ice-state.info, Chapter 3) associated with recent IS2 winter thickness assessments (Petty and others, 2023c).

**Figure 4.**
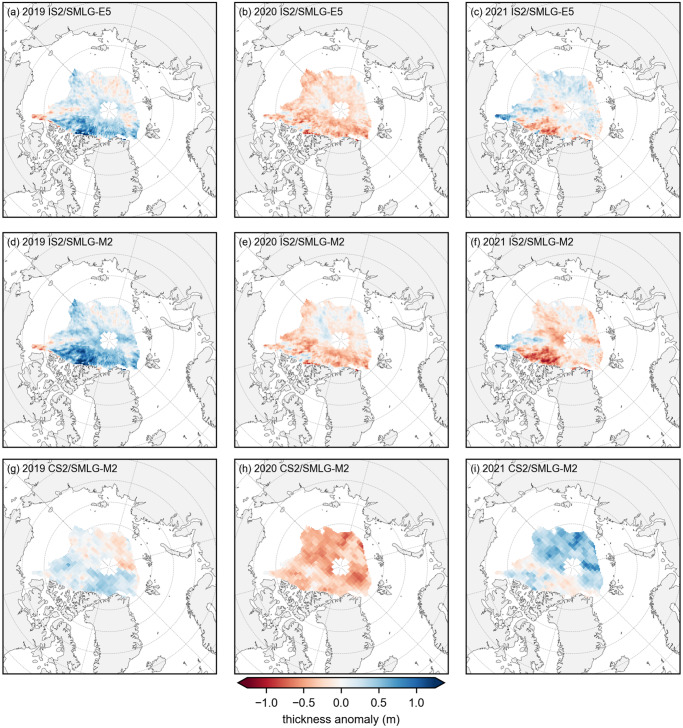
Summer mean (May to August) sea ice thickness anomalies relative to the three year (2019 to 2021) summer means for (a–c) ICESat-2 and ERA5 forced SnowModel-LG (IS2/SMLG-M2); (d–f) ICESat-2 and MERRA-2 forced SnowModel-LG (IS2/SMLG-M2); (g–i) CryoSat-2 and SMLG-M2 (CS2/SMLG-M2). The monthly data only include grid-cells where both datasets show consistent monthly data across both datasets (a perennial common mask) before averaging. The 2019 means do not include July (missing ICESat-2 freeboards), while 2021 means do not include August (no available SMLG data).

Figure 4 shows the summer thickness anomalies relative to the three-summer mean for IS2/SMLG-E5, IS2/SMLG-M2 and CS2/SMLG-M2. The three estimates show some encouraging agreement in the inter-annual summer thickness anomaly patterns, e.g. the broad negative anomaly in the 2020 summer and the large-scale regions of competing negative/positive anomalies in 2019, but do show bigger disagreements in summer 2021 (May through July only). The IS2/SMLG-E5 spatial anomalies are slightly weaker than IS2/SMLG-M2 overall and show slightly better agreement with CS-2/SMLG-M2 anomalies, especially in summer 2021. Despite the use of a common 25 km grid size in this analysis, the native resolution of the CS2/SMLG-M2 data is 80 km, which is one likely reason why the CS2 anomalies show less grid-scale variability than IS2.

**Figure 5.**
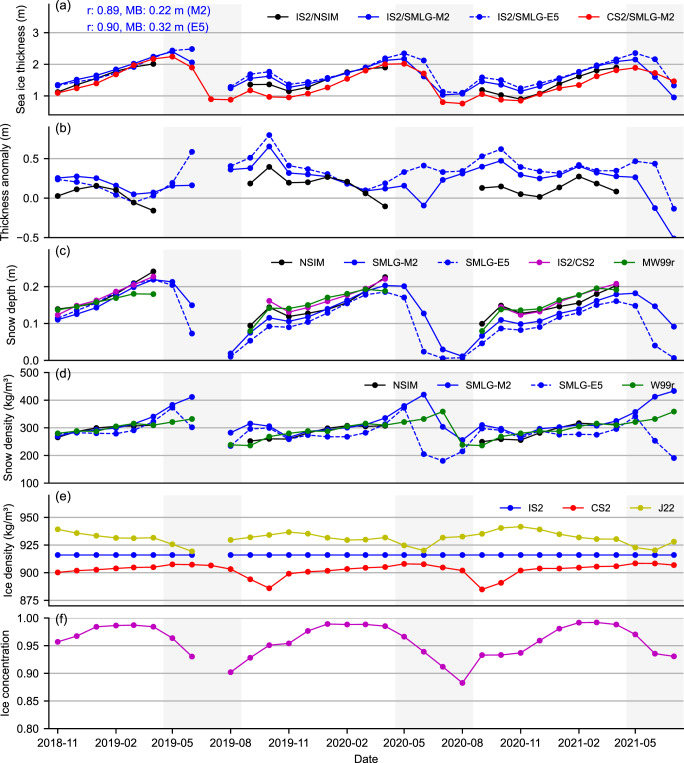
Three years (November 2018 to July 2021) of (a) monthly Arctic sea ice thickness from IS2SITMOGR4 v4 with NESOSIM v1.1 snow loading (NSIM, winter), ICESat-2 with MERRA-2 and ERA5 forced SnowModel-LG snow loading (SMLG-E5 and SMLG-M2, all-season) and CryoSat-2 with SMLG-M2 (CS2/SMLG-M2, all-season); (b) thickness anomalies relative to CS2/SMLG-M2; (c) snow depths from NSIM, SMLG-M2 and SMLG-E5 (sub-sampled/gridded by IS2), dual altimetry fusion snow depths (IS2/CS2), the modified/regional Warren snow climatology (MW99r); (d) snow density from NSIM, SMLG-M2, SMLG-E5 and W99; (e) bulk ice density assumptions used in IS2 (916 fixed), CS2 (ice type weighting) and the J22 parameterization using IS2/SMLG-M2 data; (f) ice concentration from passive microwave (monthly NSIDC CDR v4 data). All data are first masked outside of an Arctic Ocean region shown in Fig. 1, and an additional common masking is applied each month, with monthly grid-cells masked if not included in both IS2/SMLG and CS2/SMLG-M2 datasets for the given month. No July 2019 estimates are provided for IS2 as detailed in Section 2.1. Gray shading highlights the summer months (May to August).

Figure 5 shows the seasonal time-series comparison of Arctic sea ice thickness from both IS2 and CS2 all-season thickness estimates, as well as the winter (September to April) IS2 thickness based on NESOSIM v1.1 snow loading (IS2/NSIM, IS2SITMOGR4). In this analysis, we use all data within an Arctic Ocean region shown in Fig. 1 and a common (not perennial) masking to ensure each month we only use grid-cells where both IS2 and CS2 products provide valid data across all variables. Figure 5b also shows the monthly sea ice thickness anomalies of the two IS2/SMLG thickness estimates relative to CS2/SMLG-M2. Associated metrics of monthly mean snow depth, snow density, bulk ice density and ice concentration from various sources as noted are given in panels c to f. Overall, the IS2/SMLG-M2 and CS2/SMLG-M2 all-season products show good agreement in terms of the seasonal cycle (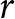 = 0.89), but with IS2/SMLG-M2 thicker than CS2/SMLG-M2 for most of this common time-period shown (mean bias of 22 cm). Both products capture a slight thinning between September and November as FYI begins to form and coverage expands across our Arctic Ocean study region (note the concentration increase in panel f). They also both show consistent thickening between November and April/May and a thickness decline between May or June (depending on the year and product) and August. IS2/SMLG generally exhibits a faster rate of summer thickness decline between June and July than CS2/SMLG-M2. The IS2/SMLG-E5 thicknesses show similar correlation to CS2/SMLG-M2 (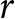 = 0.90) but with a higher mean bias of 32 cm relative to CS2/SMLG-M2.

The IS2/NSIM winter thickness estimates generally fall within the range of the IS2/CS2 SMLG-derived thickness estimates, with all thickness estimates showing the best agreement in the first winter period shown (2018-11 to 2019-04). The winter IS2/NSIM thicknesses are on average 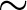
12 cm thicker than CS2/SMLG-M2, but with strong seasonal variability. While the focus of this study was incorporating the new summer (May through August) data, some of the biggest monthly differences manifest during the fall freeze-up period and should be an area of future focus. A large fraction of the sea ice is multi-year ice during fall, and a lower ice density is assumed for multi-year ice in the CS2 thickness estimates compared to the IS2 estimates, which could be a significant contributor to the differences in fall.

Differences in the spring/summer snow decline across the two SMLG estimates are clearly apparent, with SMLG-M2 showing a later and slower rate of snow loss compared to SMLG-E5, increasing the agreement with CS2/SMLG-M2. The summer SMLG snow depth differences are up to 10 cm in some months, driving thickness differences of up to 40 cm. The faster SMLG-E5 snow depth decline results in a slower IS2/SMLG-E5 thickness decline, although CS2 would see the opposite impact based on the contrasting impact of snow depth on freeboard-thickness conversion for total vs ice freeboard data. Additional analyses has suggested an overestimation of the seasonal cycle in the CS2/SMLG-M2 data (Song and others, 2024).

The strong agreement in the first winter season, and the middle of the second winter season (January to March 2020), is noteworthy for being the periods when SMLG snow depths show the best agreement with NESOSIM and the two other provided snow depth estimates (IS2/CS2 and MW99r). This provides some qualitative evidence of consistency between the IS2 and CS2 freeboard retrievals in the absence of significant snow biases. Both SMLG estimates show thinner snow overall in the subsequent two winters, which is less evident in the NESOSIM, IS2/CS2 or MW99 snow depths. The strong consistency in the NSIM and IS2/CS2 snow depth products provides some limited evidence of a negative bias in SMLG in the later part of this time-period, which coincides with months of more significant thickness disagreement. Errors in snow depths contribute in an approximately opposing manner to laser vs radar freeboard-to-thickness conversion, driving thickness differences even in the absence of any freeboard bias in either product. For example, a low snow depth bias would produce a positive IS2 thickness bias but a negative CS2 thickness bias, which we discuss more later. Significant thickness differences are also observed between the IS2 and CS2 products using the same SMLG snow loading during the non-winter months. Between May and early July there is generally still a significant snow load on the ice, so we can assume that any winter bias in the snow loading (for any product) would extend into spring and summer. However, in the late summer, the lack of snow makes it less likely this can explain the biases between IS2 and CS2 thickness estimates.

Snow density differences are clearly notable between SMLG and NESOSIM/MW99, with the two SMLG estimates showing significant differences in the summer months, linked to the different timing and rate of snow melt. SMLG-E5 also shows slightly lower snow densities in winter compared to the other products. Snow density generally provides only a second-order impact on thickness and is thus not considered a major driver of the thickness differences observed, but is worth considering in future development and inter-comparison efforts.

Finally, Fig. 5e shows the different bulk ice density assumptions used in the IS2/SMLG (fixed at 916 kg m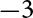) and CS2/SMLG-M2 products (weighted by ice type, lower for multiyear ice) as well as a new bulk ice density estimate based on the J22 parameterization and our IS2 freeboards, as discussed in Section 2.1. The CS2 ice type density assumption results in mean densities of 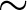
900 kg m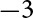 compared to the constant value of 916 kg m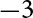 in IS2, reducing further in summer when the ice is mostly composed of remnant MYI. This density difference could also be driving a considerable fraction of the IS2/SMLG and CS2/SMLG-M2 thickness differences, if either product adopted the alternative bulk ice density assumption that would increase/decrease resultant ice thickness and reduce the overall bias by 20-30 cm on average. The J22/IS2 bulk ice density parameterization output of 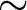
920-940 kg m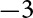 provides an alternative estimate of higher densities than the current assumptions used in either primary IS2 or CS2 thickness products, which drive higher thicknesses across both datasets. However, as stated earlier, this new bulk ice density parameterization was developed with multi-sensing airborne data collected in spring within limited campaigns across the Western Arctic only, and thus needs to be further validated across different regions and time-periods.

### 3.2. Comparisons with independent data

We next provide a series of independent data assessments to add insight into the accuracy of the various satellite-derived products, including data from Upward Looking Sonar (ULS) mooring ice draft observations from the Beaufort Gyre Exploration Project (BGEP), snow and ice thickness buoy observations deployed during the 2019–20 MOSAiC expedition, and snow and ice thickness airborne observations from the 2019 AWI IceBird campaign.

#### 3.2.1. BGEP ULS

**Figure 6.**
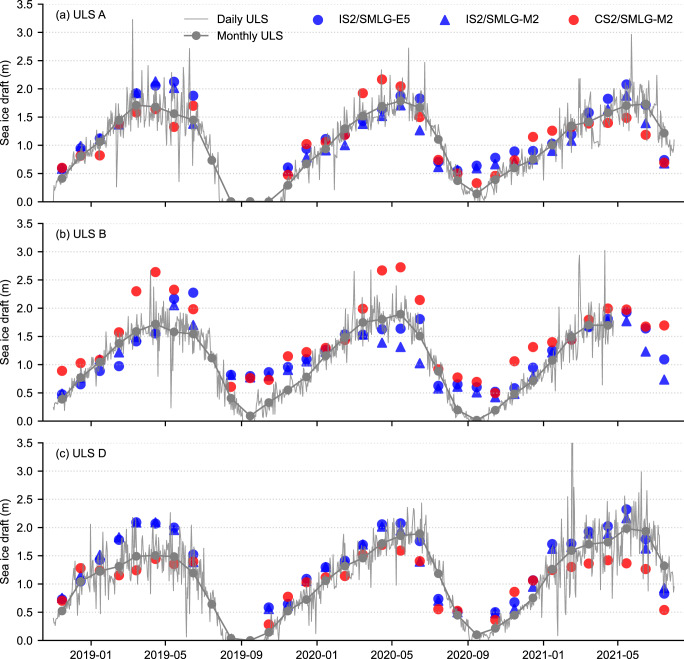
Three year time-series comparison between Beaufort Gyre Exploration Project (BGEP), Upward Looking Sonar (ULS) ice draft measurements (daily and monthly means), coincident ice drafts from ICESat-2 with ERA5 and MERRA-2 forced SnowModel-LG snow loading and CryoSat-2 with MERRA-2 forced SnowModel-LG at the three different BGEP mooring locations shown in Fig. 1.

Figure 6 shows a comparison of monthly mean BGEP ULS ice drafts with our satellite-derived ice draft estimates for IS2/SMLG-E5, IS2/SMLG-M2 and CS2/SMLG-M2 all-season derived ice drafts as described in Section 2.4. Some months in Fig. 6 are missing satellite-derived estimates due primarily to reduced summer ice coverage (the moorings are located close to the summer ice edge, IS2 and CS2 processing filters data with ice concentration below 50% and 30%, respectively). Both IS2/SMLG estimates and CS2/SMLG capture the overall seasonal cycle of ice draft and the broad regional differences across the three moorings (all still within the Beaufort Sea). However, higher IS2/CS2-derived ice drafts are clearly evident during late-summer/early months over ULS mooring B and high CS2/SMLG-derived ice drafts are observed in some winter months, also over ULS mooring B. 2021 winter/spring is also notable for showing small positive biases in IS2/SMLG vs negative biases in CS2/SMLG compared to mooring D and A to a lesser extent, the period when SMLG was highlighted previously as showing a negative snow depth bias compared to the other snow products. This again alludes to the possible role of biases in snow depth driving the biases between the satellite-derived ice drafts with the moorings, since snow depth is the only variable of the hydrostatic equation that impacts ice thickness in contrasting directions for ICESat-2 and CryoSat-2, as discussed earlier.

**Figure 7.**
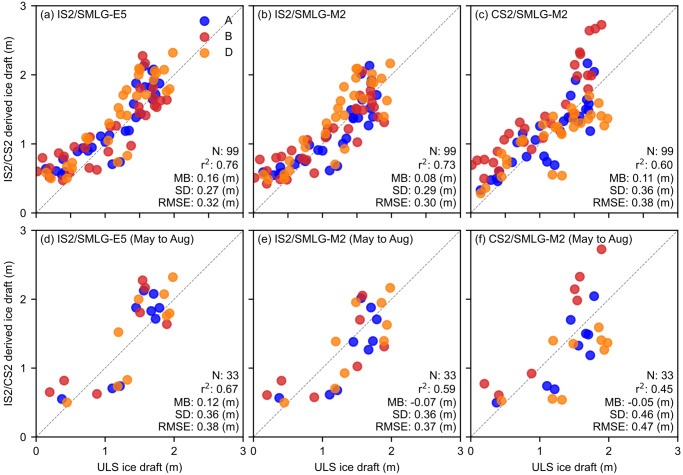
Scatter plot comparisons of monthly mean Beaufort Gyre Exploration Project (BGEP), Upward Looking Sonar (ULS) ice draft measurements and coincident (a) ICESat-2 with ERA5 forced SnowModel-LG; (b) ICESat-2 with MERRA-2 forced SnowModel-LG (SMLG-M2); (c) CryoSat-2 with SMLG-M2 derived ice drafts for the three different BGEP mooring locations from the time-series shown in Fig. 1. Panels (d), (e) and (f) show data for the May to August summer months only. Statistics show the number of coincident data points (N), coefficient of determination r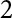, mean bias (MB), standard deviation of differences (SD) and root mean squared error (RMSE).

To better highlight the differences between the satellite-derived ice drafts and ULS ice drafts, Fig. 7a and b represents the same all-season data as scatter plots with relevant statistical metrics included. The agreement with BGEP/ULS is generally stronger for IS2/SMLG compared to CS2/SMLG-M2, especially for the IS2/SMLG-M2 data with lower root mean squared errors (RMSEs) for IS2/SMLG-M2 (0.30 m) compared to CS2/SMLG (0.38 m) and higher IS2/SMLG correlations (r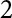 = 0.76 and 0.73) compared to CS2/SMLG (r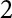 = 0.60). The mean bias (MB) is higher for IS2/SMLG-E5 (0.16 m) compared to IS2/SMLG-M2 (0.08 m) and CS2/SMLG-M2 (0.11 m). The CS2/SMLG-M2 drafts features stronger spread across the distribution, hence the larger standard deviation of differences (0.36 m compared to 0.27 m and 0.28 m for IS2/SMLG). The high correlations primarily reflect the ability of IS2/CS2 to capture the significant seasonal cycle in thickness/draft, as this is the largest signal in these data. Note that similar comparisons for CS2/SMLG over the longer 2010 to 2021 period were provided in Landy and others (2022), with the longer time period enabling an increased focus on sub-seasonal thickness skill. Additional years of IS2 data will help enable similar sub-seasonal skill assessments.

In Fig. 7d–f, we show the same BGEP/ULS comparisons for summer months only (May through August). The correlations decrease slightly for the three products, although not significantly (r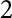 = 0.67 for IS2/SMLG-E5, 0.59 for IS2/SMLG-M2 and 0.45 for CS2/SMLG). The RMSE increases to 0.38 m (IS2/SMLG-E5), 0.37 m (IS2/SMLG-M2) and 0.51 m (CS2/SMLG) with the mean bias reducing for the three estimates including a switch to negative bias for the two M2 estimates relative to the all-season comparisons. The IS2/SMLG summer metrics are encouraging and suggest positive skill in IS2 for sub-seasonal/inter-annual summer sea ice thickness assessments. The more limited summer data availability and the presence of these moorings near to the summer ice edge means we advise caution when interpreting these results.

**Figure 8.**
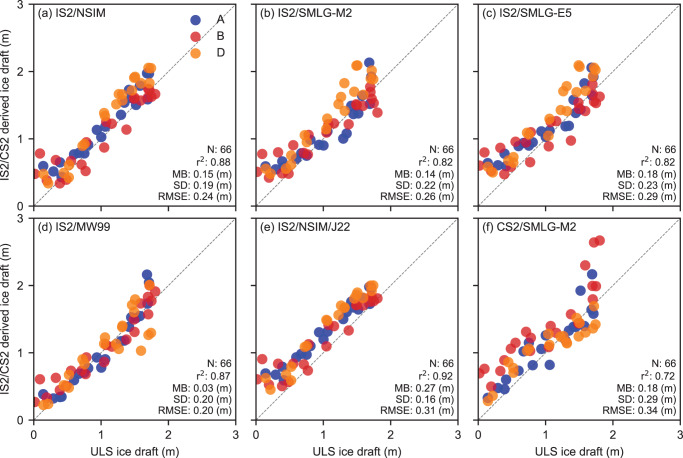
As in Fig. 7 but showing winter data (September to April) for ICESat-2-derived ice drafts with five different input assumptions, (a) NESOSIM v1.1 snow loading; (b) MERRA-2 forced SnowModel-LG snow loading (SMLG-M2); (c) ERA5 forced SMLG; (d) modified/regional Warren climatology snow loading; (e) NSIM snow loading and J22 bulk ice density; (f) CryoSat-2 with SMLG-M2.

To provide additional insight into the winter ICESat-2 thickness estimates (IS2SITMOGR4 v3/v4) and their sensitivity to our chosen input assumptions, Fig. 8 shows winter comparisons using IS2 thickness derived with different input assumptions, including MW99r snow loading and J22 ice density (with the NESOSIM v1.1 snow loading). We provide these to give more context to our assessments and to highlight the sensitivity of our results to plausible input assumptions. Currently, the MW99r snow loading and resultant thicknesses are only available in the winter IS2SITMOGR4 product due to the availability of underlying ice type data, while the J22-derived thickness are expected to be more reliable in winter, hence the more limited focus of this comparison. Note that while we call this winter, it includes data from September to April which still includes a significant seasonal cycle, driving much of the expected skill (Nab and others, 2024).

The RMSE values range from 0.20 m (IS2/MW99) to 0.34 m (CS2/SMLG-M2), with the IS2/MW99 performance notably strong (also a 0.03 mean bias). The correlations are highest for IS2/NSIM (0.88), IS2/MW99r (0.87) and IS2/NSIM/J22 (0.92). The IS2/NSIM/J22 comparisons, despite showing the highest correlations, also produce the highest mean bias (0.27 m) relative to the other estimates. The comparisons with IS2 or CS2 and SMLG snow depths show weaker correlations than for NSIM or MW99 snow loading, although all still show strong correlation (
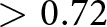
). The CS2/SMLG-M2 comparisons are slightly weaker than IS2/SMLG-M2, with a slightly larger mean bias (0.18 m compared to 0.14 m) and RMSE (0.34 m compared to 0.26 m), driven in part by a few anomalously high ice drafts over the northernmost Mooring B. All the satellite-derived ice draft estimates struggle to capture the thin ice drafts, especially the IS2/SMLG and IS2/NSIM data, suggesting possible issues with positive snow depths biases over thinner ice in the fall freeze-up period, as was also shown in Fig. 6.

#### 3.2.2. AWI IceBird-2019

**Figure 9.**
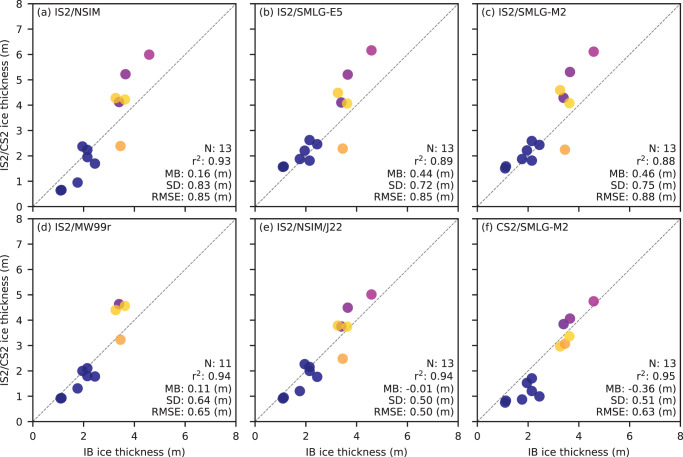
Comparison of IceBird airborne ice thickness estimates in April 2019 (binned to a 100 km North Polar Stereographic grid) and coincident monthly mean 100 km coarsened ice thickness from ICESat-2 with five different input assumptions (a) NESOSIM v1.1 snow loading; (b) ERA5 forced SnowModel-LG (SMLG) snow loading; (c) MERRA-2 forced SMLG snow loading; (d) modified/regional Warren snow loading; (e) NSIM snow loading and J22 bulk ice density; and (f) CryoSat-2 with MERRA-2 forced SMLG snow loading. Panel (f) in Fig. 10 shows the IceBird 2019 flight-lines color-coded by longitude, which are used in the colors across all scatter plots.

In Fig. 9, we compare April 2019 IceBird thickness estimates with coincident monthly mean thickness from ICESat-2 using five different input assumptions as well as CryoSat-2/SMLG-M2. In this analysis, the data are binned or coarsened to the 100 km x 100 km North Polar Stereographic grid as discussed in Section 2, resulting in only 13 grid-cells for comparison. 25 km versions are available in the Supplementary Material (Figs. S6 and S7), showing similar or worse comparison metrics overall. All products reproduce the large-scale spatial gradients observed during IceBird-2019, with correlations high across all products (r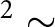 0.88). IS2/NSIM/J22 results in the lowest mean bias (–0.01 m) and RMSE (0.50 m) of all thickness estimates, however this is not a truly independent validation as the J22 ice density parameterization was developed in part using the same IceBird data presented here. The CS2/SMLG-M2 product shows good agreement over the thicker ice north of Greenland/Canadian Arctic, but a negative bias over the thinner ice profiled by IceBird-2019 over the Beaufort Sea. The IS2 products tend to exhibit positive thickness bias in the thicker ice north of Greenland/Canadian Arctic, but show better agreement in the thinner ice of the Beaufort Sea.

**Figure 10.**
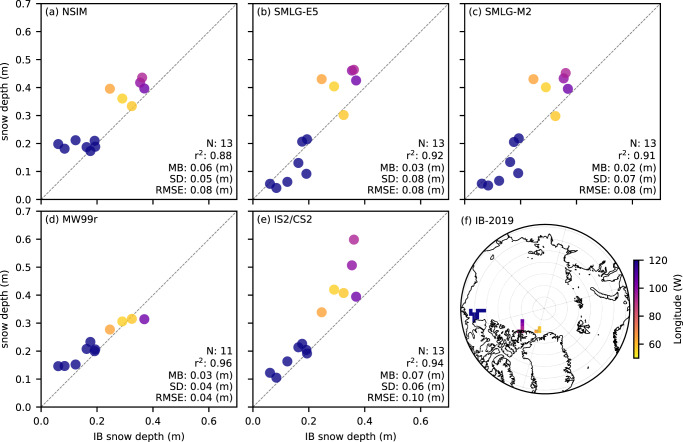
Comparison of IceBird airborne snow depth estimates in April 2019 (binned to a 100 km North Polar Stereographic grid) and coincident monthly mean coarsened snow depths (sub-sampled by ICESat-2) from (a) NESOSIM v1.1 snow loading; (b) ERA5 forced SnowModel-LG snow loading, (c) MERRA-2 forced SnowModel-LG snow loading; (d) modified/regional Warren snow loading; (e) dual altimetry fusion snow depths. Panel (f) shows the IceBird-2019 flight-lines color-coded by longitude, which are used in the colors across all scatter plots.

In Fig. 10, we compare the IceBird-2019 derived snow depths and five snow products, NSIM, SMLG-E5, SMLG-M2, MW99r and the preliminary IS2/CS2 dual altimetry fusion estimates. The correlations are again very high (r
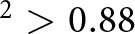
), with mean biases ranging from 0.02 (SMLG-M2) to 0.07 (IS2/CS2) and RMSE values ranging from 0.04 m (MW99r) to 0.10 m (IS2/CS2). The performance of the MW99r snow depths (and resultant sea ice thickness to a lesser extent, as shown in Fig. 9d) is notably high, however the snow depths do not capture the full-range of snow conditions observed by IceBird-2019 (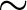
15 to 32 cm for MW99 compared to 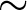
4 to 40 cm for IceBird) which we might expect from a climatology product. The IceBird-2019 flights cover regions expected to be broadly representative of the MW99 regional distribution based on the original station locations (Warren and others, 1999). While SMLG and NSIM were able to simulate higher snow depths north of the Canadian Arctic, only the SMLG-derived snow depths simulated the lower snow depths observed by IceBird-2019 over the Beaufort Sea. The IS2/CS2 dual altimetry fusion snow depths show encouraging performance overall, especially at the lower end of the distribution, but include a few abnormally high snow depths in the region north of Greenland/Canadian Arctic, which reduce overall performance. This could be caused by biases in either or both the IS2 and CS2 freeboard observations; however, the results do not point toward partial penetration of the CS2 Ku-band radar signal into the snow, which would lead to underestimated satellite snow depths.

#### 3.2.3. MOSAiC SIMBA buoys

**Figure 11.**
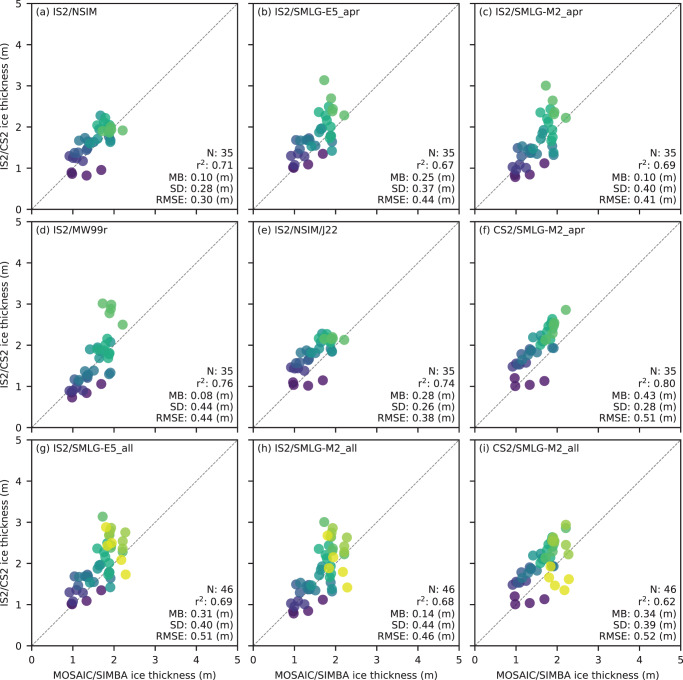
Comparisons of October 2019 to July 2020 MOSAiC/SIMBA buoy ice thickness measurements (binned to a 100 km North Polar Stereographic grid) and coincident monthly mean coarsened ice thickness estimates from ICESat-2 with (a) NESOSIM v1.1 (NSIM) snow loading through April 2020; (b) ERA5-forced SnowModel-LG snow loading (SMLG-E5) through April 2020; (c) MERRA-2 forced SnowModel-LG (SMLG-M2) snow loading through April 2020; (d) modified/regional Warren99 snow loading through April 2020; (e) NSIM snow loading and J22 bulk ice density through April 2020; (f) CryoSat-2 with SMLG-M2 through April 2020; (g) ICESat-2 and SMLG-E5 snow loading through June 2020; (h) ICESat-2 and SMLG-M2 snow loading through June 2020; (i) CryoSat-2/SMLG-M2 data through June 2020. Scatter colors based on the MOSAiC/SIMBA track shown in Fig. 12.

In Fig. 11, we compare the MOSAiC/SIMBA buoy-derived thicknesses with coincident monthly mean thicknesses from ICESat-2 using five different input assumptions as well as CryoSat-2/SMLG-M2. The data are again binned or coarsened to the 100 km x 100 km North Polar Stereographic grid. The higher resolution (25 km) versions are available in the Supplementary Material (Figs. S8 and S9), showing similar/worse comparison metrics. Here, the data are also delineated into October to April ‘apr’ comparisons based on the availability of auxiliary data with different input assumptions, and the full October to June ‘all’ MOSAiC/SIMBA data range (for both SMLG-based thickness estimates), resulting in 35 and 46 grid-cells for comparison, respectively. Overall, all products show good skill in capturing the combined seasonal and regional variability in ice thickness along the MOSAiC/SIMBA track, with correlations generally strong across all products (r
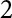
=0.67 to 0.80). The RMSEs are relatively low, ranging from 0.30 m (IS2/NSIM) to 0.52 m (CS2/SMLG-M2_all), with mean biases of 0.08 m (MW99r) to 0.43 m (IS2/SMLG_apr). The introduction of the May/June data does not cause any notable impact on the comparison metrics. Unlike the previous analyses, the use of J22 ice densities does not result in improved agreement (the RMSE instead increases from 0.30 m to 0.38 m). The two IS2/SMLG derived thicknesses show similar comparison statistics, but with again slightly improved performance in the M2 forced SMLG results.

**Figure 12.**
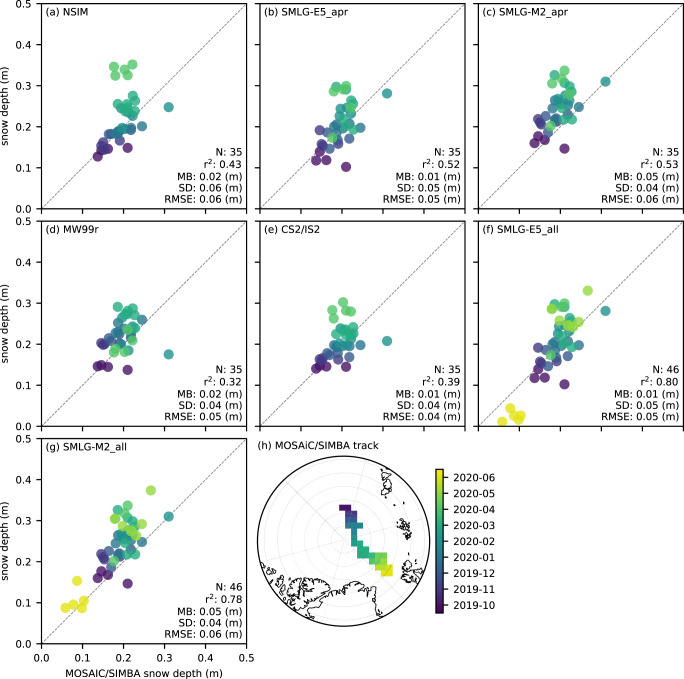
Comparisons of October 2019 to June 2020 MOSAiC/SIMBA buoy snow depth measurements (binned to a 100 km North Polar Stereographic grid) and coincident monthly mean coarsened snow depths (sub-sampled by ICESat-2 and redistributed before gridding) from (a) NESOSIM v1.1 snow loading (NSIM) through April 2020; (b) ERA5 forced SnowModel-LG (SMLG-E5) snow loading through April 2020; (c) MERRA-2 forced SMLG snow loading through April 2020; (d) modified Warren 99 snow loading (MW99) through April 2020; (e) IS2/CS2 dual altimetry fusion snow depths; (f) SMLG-E5 snow loading through June 2020; (g) SMLG-M2 snow loading through June 2020. Panel (h) shows the MOSAiC/SIMBA track color-coded by date, which is used in the colors across all scatter plots.

In Fig. 12, we show comparisons between the MOSAiC/SIMBA buoy-derived snow depths and the five snow products, NESOSIM v1.1, MW99, IS2/CS2 and both SnowModel-LG estimates for the same October to April time-period and the full October to June overlapping period. For the October to April comparisons, the five products show broadly similar results, with moderate correlations (r
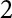
=0.32, MW99r to 0.53, SMLG-M2_apr), and low RMSEs (0.04 m to 0.06 m). The mean bias is however higher for SMLG-M2_apr (0.05 m) compared to SMLG-E5_apr and the other products despite the higher correlation. The strong performance of the MW99r climatology is again notable, especially as the MOSAiC/SIMBA buoy track includes more of the Eastern/North Atlantic Arctic sector, further outside the focal region of the station data used to compile the climatology. The IS2/CS2 dual altimetry fusion product also performs well, featuring the lowest RMSE of all four products. The NSIM, IS2/CS2 and SMLG products show higher snow depths in April (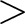
30 cm) compared to MOSAiC/SIMBA (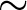
20 cm), which appears to dominate the reduction in correlation. The representativeness of the MOSAiC/SIMBA buoy data and their utility for characterizing biases are still questionable, which we discuss more later. The earlier time series analysis (Fig. 5) also highlighted the strong consistency between NSIM, MW99r and the IS2/CS2 snow depth estimates in the October 2019 to March 2020 period, but with MW99r declining in April 2020 and NSIM/IS2/CS2 increasing. Finally, the thinner snow depths in May/June appear well-represented in both SMLG datasets (albeit with a slight negative bias in the E5 forced data), resulting in a significant increase in correlation (r
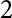
=0.79) while other metrics remain constant, providing some good, albeit limited, evidence of SMLG simulating the summer snow depth decline. It is again important to recognize that buoy-derived snow depths are prone to representation issues and are particularly uncertain during the summer melt season.

## 4. Discussion

Our new ICESat-2 all-season thickness estimates perform well overall, showing good agreement with the CryoSat-2 all-season product and strong performance across the three independent validation data assessments. We explored the use of both the ERA5 (SMLG-E5) and MERRA-2 (SMLG-M2) forced SnowModel-LG snow loading in our all-season ICESat-2 thickness estimates. The two estimates exhibit important differences in the seasonal cycle, including significant differences in the timing and rate of the spring/summer snow declines (SMLG-E5 with earlier/faster declines) and significant (up to 40 cm) impacts on sea ice thickness. The missing IS2 in July 2019 prevented a more detailed interannual assessment of the summer snow decline, but should form the basis of future work. Unsurprisingly, using the same SMLG-M2 snow loading improved the agreement between ICESat-2 and the all-season CryoSat-2 product, however the relative benefits of either SMLG product based on our BGEP/ULS, IceBird-2019 and MOSAiC/SIMBA comparisons were less clear. The differences in the timing and rate of seasonal snow loss are expected to be driven primarily by differences in near-surface air temperature differences between the reanalyses, which is a known challenge where prescribed sea ice conditions/missing snow cover drive significant biases in air temperature (Batrak and Müller, 2019). Both SMLG snow depths/densities and resultant sea ice thicknesses are included in an updated Version 4 winter Arctic sea ice thickness dataset (Petty et al., 2025) and a new Version 1 summer thickness dataset (Petty, 2025) to enable future inter-comparison efforts.

While mid-late summer snow is generally thin, it can still be highly variable based on synoptic-scale variability (Webster and others, 2019; Lim and others, 2022), and even small differences in snow depth can have a disproportionately large impact on thin ice freeboard and thickness retrievals. July also generally coincides with the period of peak melt pond coverage, depending on region (Niehaus and others, 2025), so issues with misclassified surfaces in ICESat-2 sea ice data (Tilling and others, 2020) and resultant biases in ice freeboard will also be contributing to anomalous freeboard and thickness reduction and are worth more consideration. The role of potential biases in spring/summer snow melt, melt water load and impacts on CryoSat-2 derived freeboard and thickness estimates was recently discussed in Salganik and others (2025).

Snow depth biases are likely contributing to a significant fraction of the thickness biases observed between our IS2 and CS2 thickness estimates. The BGEP ULS comparisons, for example, produced periods of contrasting bias across the IS2 and CS2 datasets, which are expected because a snow depth bias will theoretically impact the estimated thickness obtained from CryoSat-2 radar freeboard and ICESat-2 total freeboard observations in opposing directions. For radar and ice freeboard to thickness calculations, the snow depth bias has a strong inverse linear relation to resultant ice thickness bias. For laser and total freeboard to thickness calculations, the impact of a snow depth bias is more complex, as a snow depth bias features in the hydrostatic equilibrium equation in both the freeboard and snow loading component, generally resulting in a thickness bias in an opposite direction, although this depends on the magnitude of the underlying snow density. This is the only variable that results in this contrasting input data bias response. The strong agreement between NESOSIM, MW99r and IS2/CS2 altimetry fusion snow depths is suggestive of a potential low bias in the SMLG snow depths in 2020 and especially 2021. However, quantifying the relative impacts of snow biases is challenging, and it is still possible that the three former products are all biased thick. The use of different bulk ice density estimates between the IS2 and CS2 could also contribute significantly to the 20 cm mean thickness difference between IS2/SMLG-M2 and CS2/SMLG-M2. Using the same density approximation would not necessarily produce more accurate absolute thicknesses, but would help isolate and diagnose remaining sources of bias.

The snow depths from the new preliminary winter Arctic IS2/CS2 altimetry fusion dataset (Landy and others, 2024) performed well in our independent validation and showed strong consistency with NESOSIM and MW99 across the three winter periods of overlap. The strong performance of this winter dual altimetry fused snow depth estimate provides further indirect evidence of the reliability of the underlying IS2 and CS2 freeboards and the potential to infer snow depth and thickness concurrently (Kacimi and Kwok, 2022; Fredensborg Hansen and others, 2024; Landy and others, 2024; Carret and others, 2025). No clear bias could be detected in the dual-altimetry snow depths that would point to systematic biases in either IS2 or CS2 freeboards. A slight thick bias relative to the IceBird-2019 observations suggests that CS2 returns are not, at least, scattering from a mean height within the snowpack, although there was only 1 cm bias recorded at the MOSAiC/SIMBAs. It should, in theory, be possible to expand these approaches to summer, leveraging the year-round ICESat-2 freeboards and recent summer CryoSat-2 ice freeboard profiling advances (Dawson and others, 2022).

The modified Warren snow climatology still performs surprisingly well, showing the lowest RMSEs in BGEP ULS ice drafts when combined with IS2 freeboards and high skill in capturing the regional distribution in snow depths observed during IceBird-2019 and MOSAiC/SIMBA (to a lesser degree). The MOSAiC/SIMBA buoys are known to have a low snow thickness bias relative to nearby magnaprobe measurements, possibly as a consequence of the deployment over level ice being more conducive to snow drifting (Lei and others, 2022). Snow conditions in the Central Arctic/Beaufort Sea appear to remain closely linked to variability in ice type (first-year or multiyear ice), which the ice type modified climatology is still principally constrained by. Furthermore, this suggests that large-scale regional and interannual variations in satellite-observed laser or radar freeboard, rather than snow loading, are more important for accurate detection of the same variations in sea ice thickness. The snow accumulation models (NESOSIM v1.1 and SMLG) and resultant sea ice thicknesses perform well across all validation datasets and better capture the full range in observed snow conditions, but struggle to show consistently more reliable snow depths than MW99 between regions or years. More validation data is needed across different time-periods and especially within the eastern Arctic, where the accumulation models are expected to provide more benefits over MW99 for multiple reasons: (i) these regions provided significantly less input data to MW99, (ii) they have undergone the largest transitions in sea ice and likely snow conditions in recent decades (Petty and others, 2018; Stroeve and Notz, 2018; Cabaj and others, 2025) and (iii) these regions are more susceptible to synoptic-scale snowfall variability, especially in summer (Webster and others, 2019; Lim and others, 2022). Regardless, there is a clear need for further snow model development and calibration efforts.

In addition, there is an urgent need for expanded year-round validation efforts, particularly a greater number of mooring deployments across the Arctic to complement the limited existing datasets, and especially in the eastern and more peripheral regions of the Arctic. As well as more validation data, we also promote more consideration of spatial and temporal sampling/representativeness to more effectively compare localized validation data against the 25-100 km scales and longer aggregation timescales represented by the satellite products. We also hope to leverage external datasets in future work to better understand surface melt conditions and associated freeboard and thickness retrieval performance in more detail, e.g. isolating periods and regions of maximum/minimum melt pond coverage (Niehaus and others, 2025). New data-driven algorithms trained on coincident satellite imagery show promise for automated discrimination of leads and melt ponds and can hopefully form the basis of future freeboard enhancement and associated validation efforts (Liu and others, 2025).

Our focus in this initial all-season assessment was on monthly to inter-annual thickness skill, but future work could explore daily to weekly skill, which should again include deeper consideration of optimal spatial and temporal sampling of the variable IS2/CS2 orbit cycles. Orbit aliasing was still present in our interpolated/smoothed IS2 June gridded thickness estimates, motivating the need for improved interpolation routines. Along-track IS2 thickness data are available (Petty and others, 2022) that can enable more direct comparisons with CS2 and reducing gridding/resampling error, especially leveraging the increased orbit alignments generated through the Cryo2Ice initiative (Fredensborg Hansen and others, 2024). In addition, more sophisticated altimetry interpolation routines have been proposed that could be better leveraged to enhance grid-cell representativeness across scales (Gregory and others, 2021).

Finally, the lack of public SM-LG data beyond July 2021 provided a clear limitation of this study and the production of our all-season IS-2 thickness data. In response to this, work is underway to produce all-season NESOSIM snow loading through inclusion of a basic melt parameterization scheme, while also leveraging recent work on NESOSIM model calibration with Markov Chain Monte Carlo (MCMC) approaches (Cabaj and others, 2023; 2025). Our hope is that the results from this study can provide a needed baseline to assess future snow loading and ice thickness production efforts.

## 5. Conclusion

We have presented an initial analysis of all-season ICESat-2 Arctic sea ice thickness by combining ICESat-2 freeboards with all-season SnowModel-LG snow loading. Our assessments show that the ICESat-2 thickness retrievals capture key regional and seasonal patterns of Arctic sea ice variability and agree well with recently published CryoSat-2 all-season estimates. Regional differences remain, with IS2 generally showing thicker ice north of Greenland and the Canadian Arctic Archipelago, which was highlighted in the original development studies (Dawson and others, 2022; Landy and others, 2022) as an uncertain region for CryoSat-2 summer observations. However, these summer differences are largely consistent with the winter differences, suggesting only secondary impacts of summer conditions impacting the biases between the two products. Validation against three different independent datasets, including BGEP ULS moorings, IceBird-2019 airborne surveys and MOSAiC/SIMBA buoy observations, highlights generally good skill across a range of ice conditions, although some regional and seasonal biases remain.

The choice of snow model inputs and ice density assumptions continues to have a strong influence on absolute thickness estimates. Differences between ICESat-2 and CryoSat-2 thicknesses are consistent with expected sensitivities to snow depth biases, freeboard retrieval differences and the underlying treatment of multi-year versus first-year ice and resultant density estimates. Some additional concerns relating to the timing and magnitude of the spring/summer snow depth decline and expected impact on thickness decline require further investigation.

While our results are encouraging, they also emphasize the need for further work to better constrain spring/summer snow conditions, refine the ICESat-2 freeboards and freeboard-to-thickness input assumptions and expand independent evaluation datasets and analysis across Arctic regions and time-periods. Continued development of snow accumulation models and associated forcings, new density parameterizations and data fusion approaches will be key to improving all-season Arctic sea ice thickness retrievals. Importantly, if satellite-derived thickness estimates are to enhance or benchmark state-of-the-art seasonal-to-climate prediction systems, they need to accurately capture inter-annual variability at regional scales of interest. While our results are promising, a longer record and further evaluation against independent observations are needed to better characterize and reduce potential biases through the freeboard-to-thickness processing chain.

## Supporting information

Petty et al. supplementary material Petty et al. supplementary material
