# Supplementary material for: Initial assessment of all-season Arctic sea ice thickness from ICESat-2
Source: J Glaciol. 2025 Dec 26;72:e4. doi: 10.1017/jog.2025.10119 (PMC12926744; doi:10.1017/jog.2025.10119)
Supplement: Petty et al. supplementary material [file S0022143025101196sup001.pdf]

Supplementary Material for "Initial assessment of all-season Arctic sea ice thickness from ICESat-2"

Alek PETTY[1], Alex CABAJ[2], Jack LANDY[3]

[1] *Earth System Science Interdisciplinary Center, University of Maryland, MD, USA*

[2] *Environment and Climate Change, Toronto, Canada*

[3] *UiT The Arctic University of Norway, Tromsø, Norway*

Correspondence: Alek Petty <akpetty@umd.edu>

CONTENTS

Fig. S1: IS2SITMOGR4S browse image for June 2020 with ERA5 forced SnowModel-LG snow loading (SMLG-E5).

Fig. S2: IS2SITMOGR4S browse image for June 2020 with MERRA-2 forced SnowModel-LG snow loading (SMLG-M2).

Fig. S3: Monthly (May through August) CS2/SMLG-M2 sea ice thickness from the UBRIS dataset.

Fig. S4: Monthly (May through August) difference maps between IS2/SMLG-M2 and CS2/SMLG-M2.

Fig. S5: Winter mean (January to April) differences between IS2/SMLG-M2 and CS2/SMLG-M2.

Fig. S6: AWI IceBird-2019 thickness comparisons, as in Fig. 9 of the main manuscript but for 25 km grid spacing instead of 100 km.

Fig. S7: AWI IceBird-2019 snow depth comparisons, as in Fig. 10 of the main manuscript but for 25 km grid spacing instead of 100 km.

Fig. S8: MOSAiC/SIMBA thickness comparisons, as in Fig. 11 of the main manuscript but for 25 km grid spacing instead of 100 km.

Fig. S9: MOSAiC/SIMBA snow depth comparisons, as in Fig. 12 of the main manuscript but for 25 km grid spacing instead of 100 km.

FIGURES

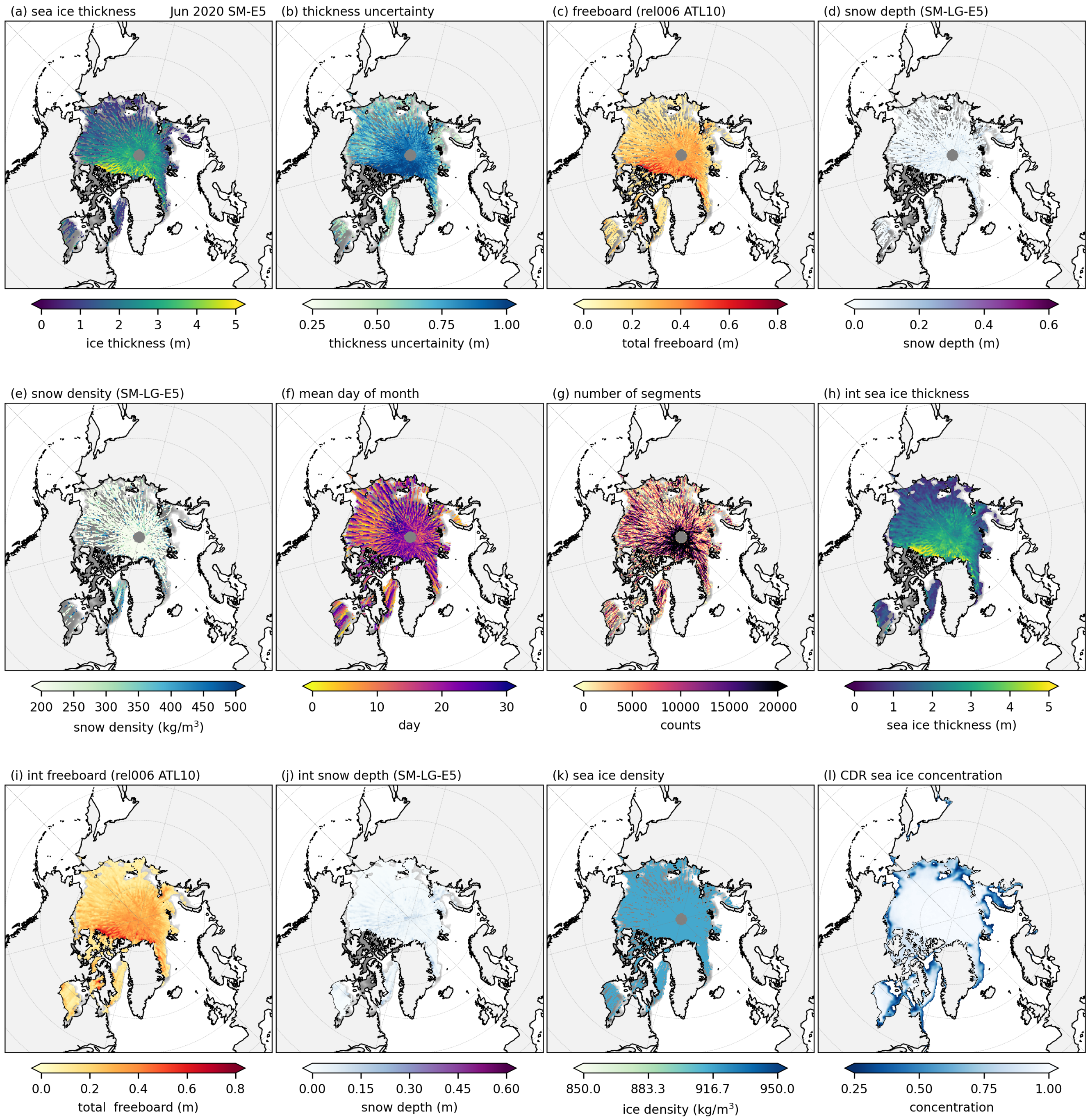

Fig. S1. IS2SITMOGR4S browse image for June 2020 with ERA5 forced SM-LG snow loading.

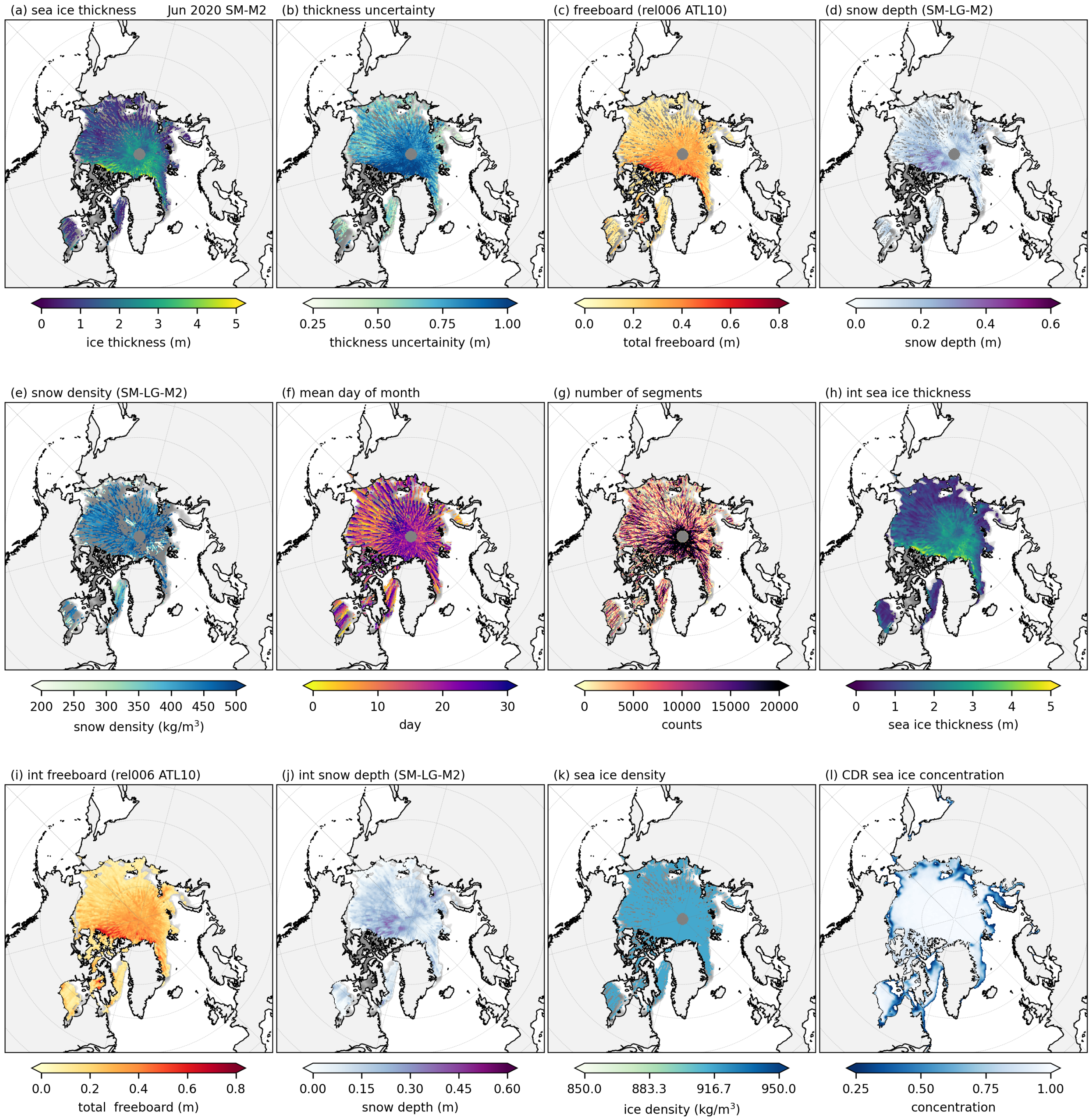

Fig. S2. IS2SITMOGR4S browse image for June 2020 with MERRA-2 forced SM-LG snow loading.

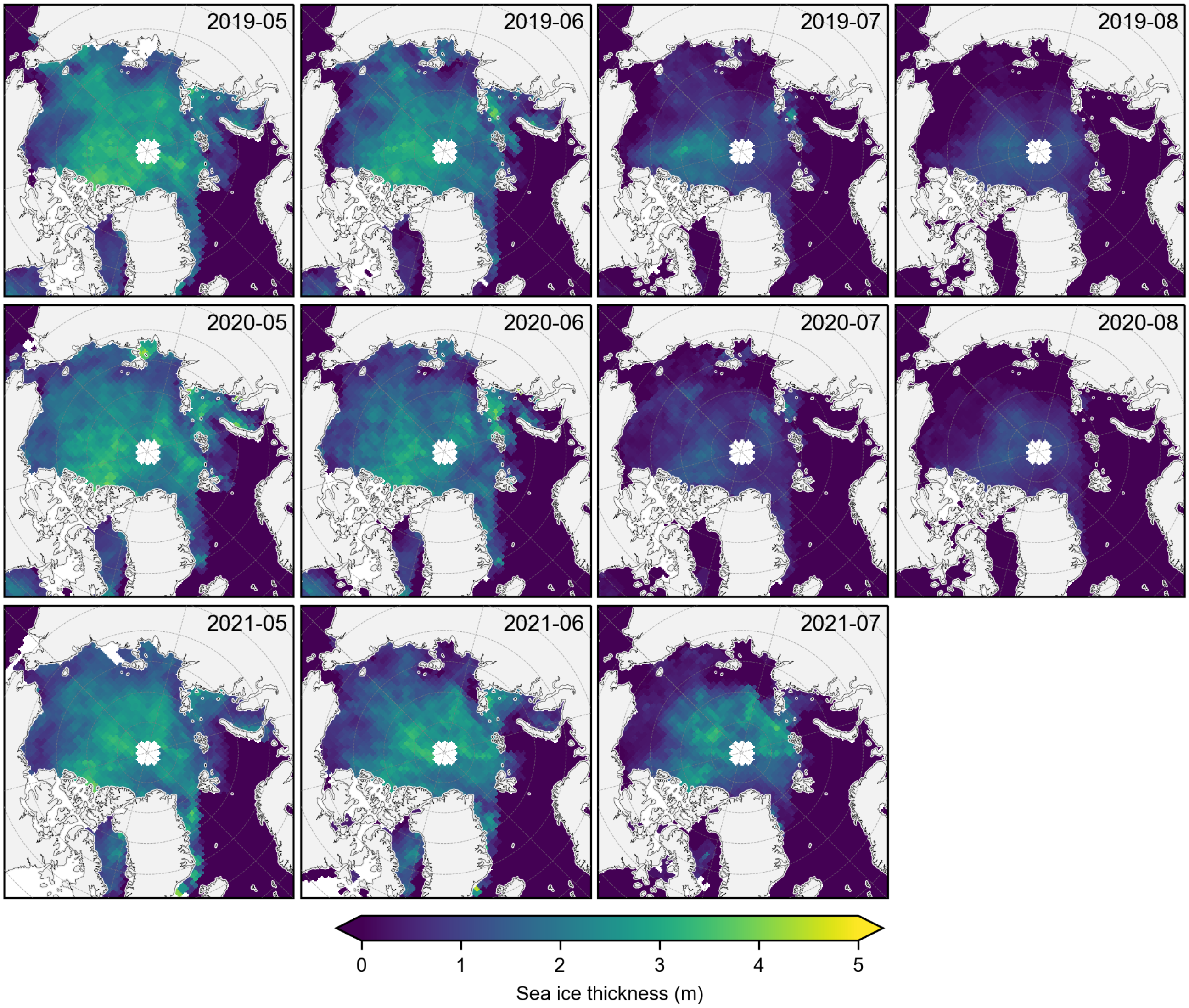

Fig. S3. Monthly mean summer (May through August, 2019 to 2021) Arctic sea ice thickness estimates from the UBRIS CryoSat-2 all-season thickness dataset.

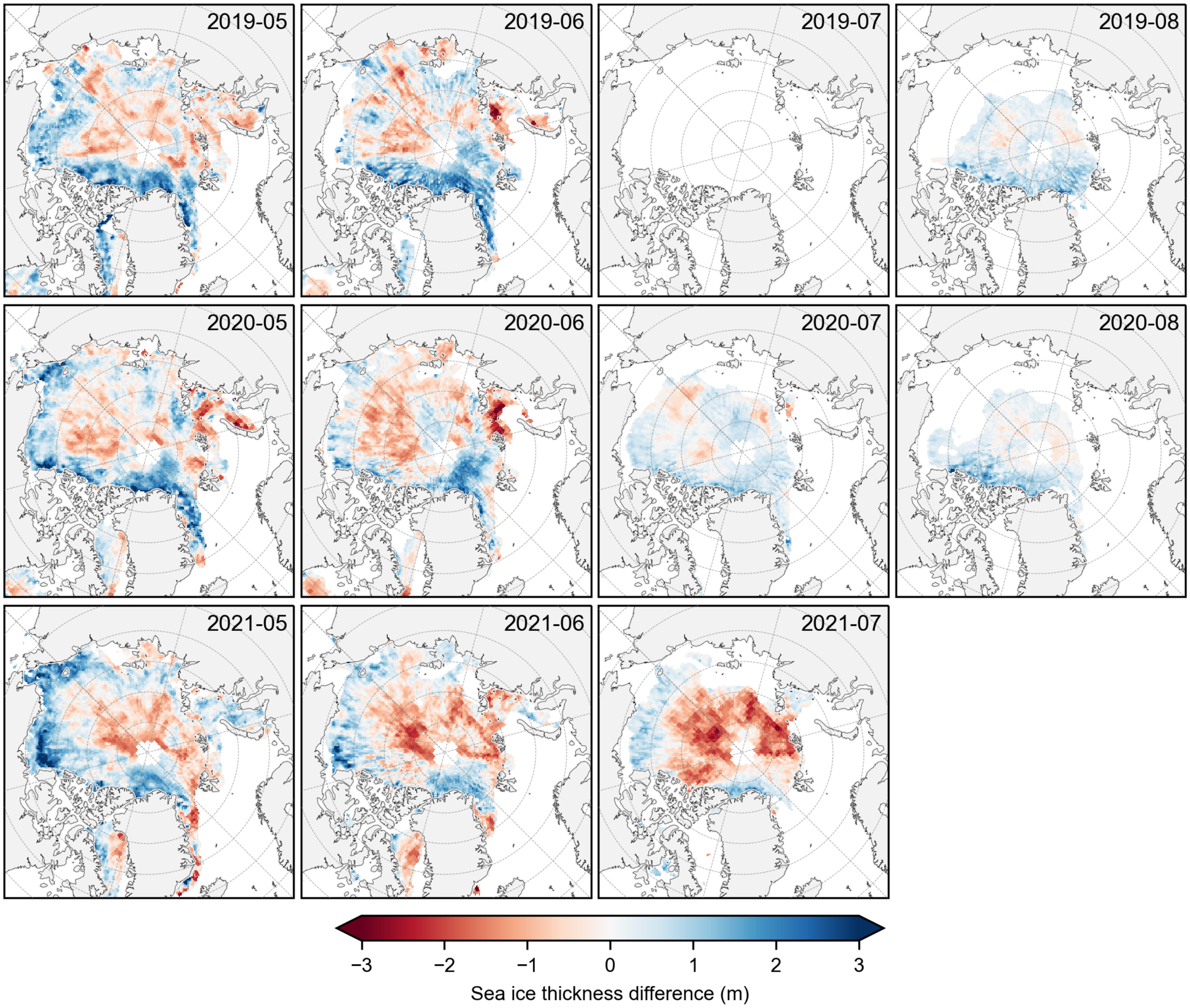

Fig. S4. Monthly mean summer (May through August, 2019 to 2021) Arctic sea ice thickness differences between ICESat-2 with MERRA-2 forced SnowModel-LG snow loading and the all-season CryoSat-2 product (UBRIS, also with SMLG-M2 snow loading).

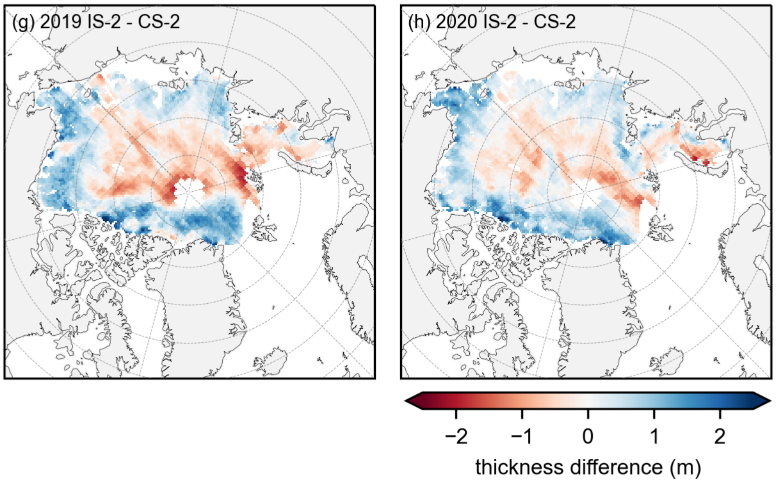

Fig. S5. Winter mean (January through April) monthly gridded sea ice thickness difference between ICESat-2 with MERRA-2 forced SnowModel-LG snow loading (IS2/SMLG-M2) and the all-season CryoSat-2 product (UBRIS, also with SMLG-M2 snow loading) for 2019 (a) 2019, (b) 2020 and (c) 2021. The monthly data are filtered where both datasets show consistent monthly data across both datasets (a perennial common mask) before averaging.

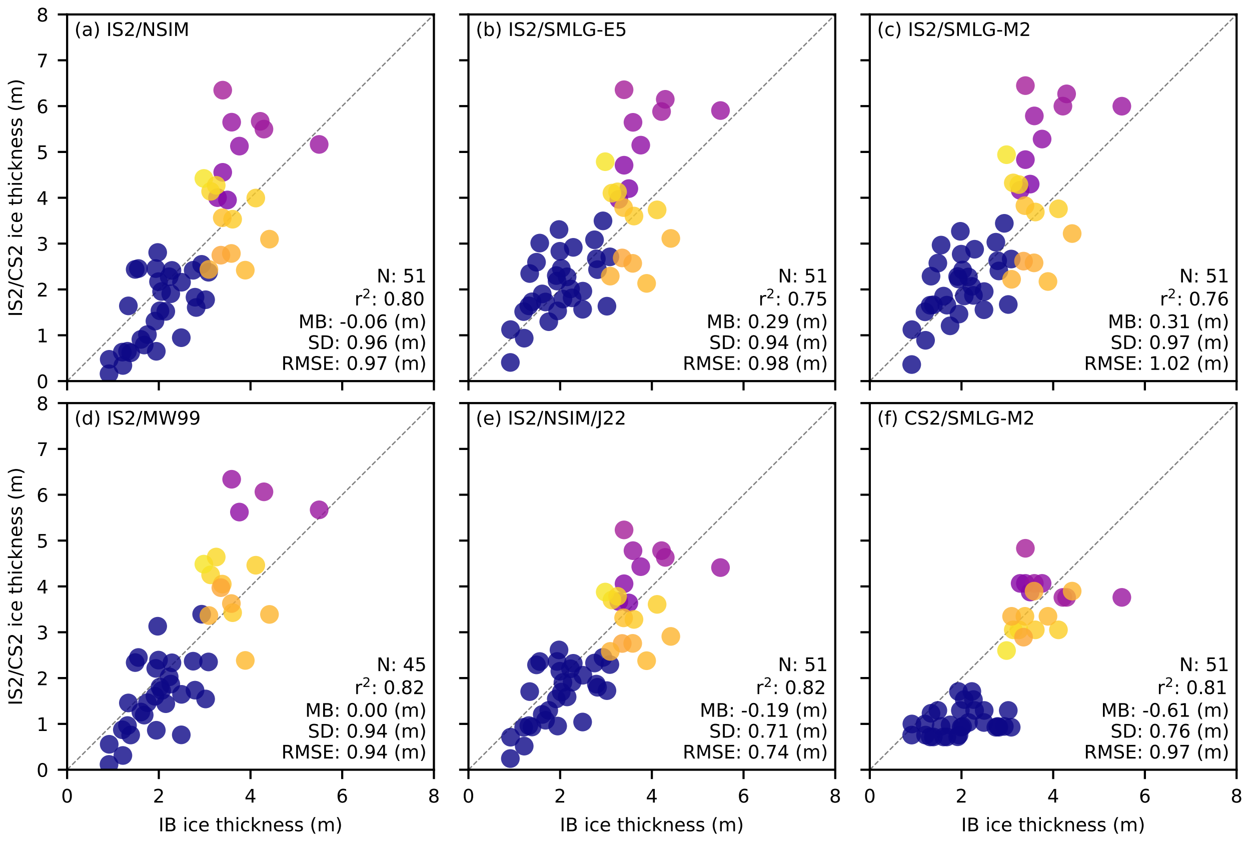

Fig. S6. Comparison of IceBird airborne ice thickness estimates in April 2019 (binned to a 25 km North Polar Stereographic grid) and coincident monthly mean 100 km coarsened ice thickness from ICESat-2 with five different input assumptions (a) NESOSIM v1.1 snow loading, (b) ERA5 forced SnowModel-LG (SMLG) snow loading, (c) MERRA-2 forced SMLG snow loading, (d) modified/regional Warren snow loading, (e) NSIM snow loading and J22 bulk ice density, and (f) CryoSat-2 with MERRA-2 forced SMLG snow loading. Panel (f) in Fig. **??** shows the IceBird 2019 flight-lines color-coded by longitude, which are used in the colors across all scatter plots.

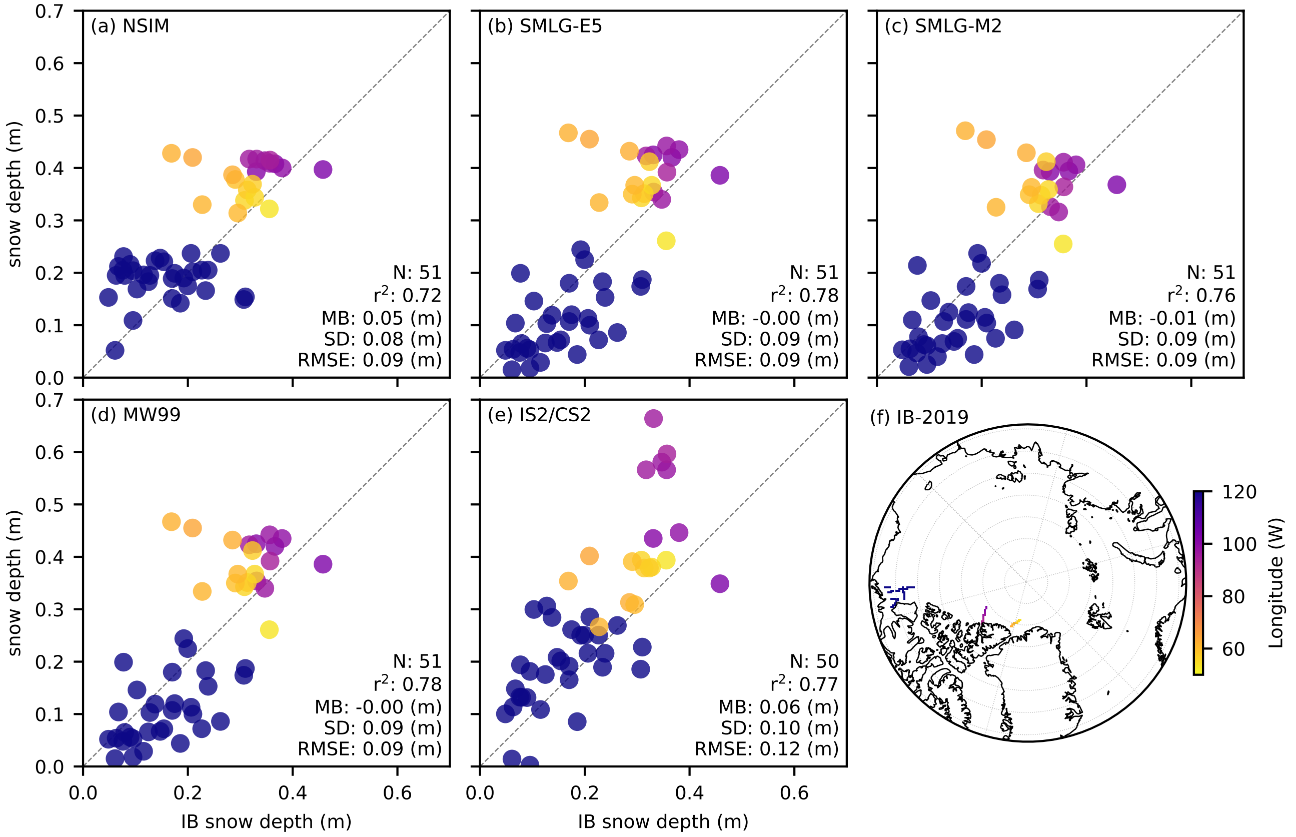

Fig. S7. Comparison of IceBird airborne snow depth estimates in April 2019 (binned to a 25 km North Polar Stereographic grid) and coincident monthly mean coarsened snow depths (sub-sampled by ICESat-2) from (a) NESOSIM v1.1 snow loading, (b) ERA5 forced SnowModel-LG snow loading, (b) MERRA-2 forced SnowModel-LG snow loading, (d) modified/regional Warren snow loading, and (e) dual altimetry fusion snow depths. Panel (f) shows the IceBird-2019 flight-lines color-coded by longitude, which are used in the colors across all scatter plots.

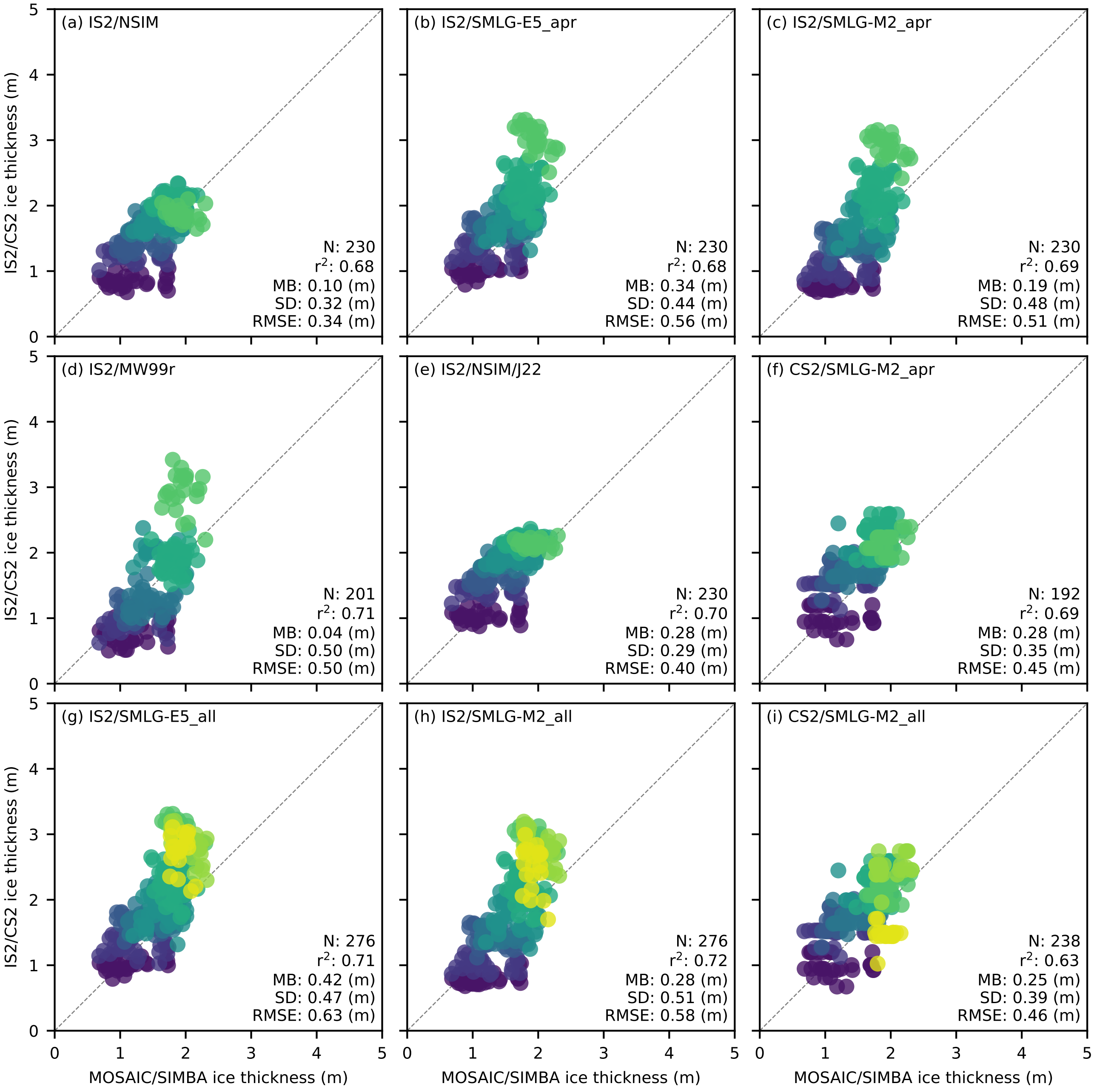

Fig. S8. Comparisons of October 2019 to July 2020 MOSAiC/SIMBA buoy ice thickness measurements (binned to a 25 km North Polar Stereographic grid) and coincident monthly mean coarsened ice thickness estimates from ICESat-2 with (a) NESOSIM v1.1 (NSIM) snow loading through to April 2020, (b) ERA5-forced SnowModel-LG snow loading (SMLG-E5) through to April 2020, (c) MERRA-2 forced SnowModel-LG (SMLG-M2) snow loading through to April 2020, (d) modified/regional Warren99 snow loading through to April 2020, (e) NSIM snow loading and J22 bulk ice density through to April 2020, (f) CryoSat-2 with SMLG-M2 through to April 2020, (g) ICESat-2 and SMLG-E5 snow loading through to June 2020, (h) ICESat-2 and SMLG-M2 snow loading through to June 2020 and (i) CryoSat-2/SMLG-M2 data through to June 2020. Scatter colors based on the MOSAiC/SIMBA track shown in Fig. S9.

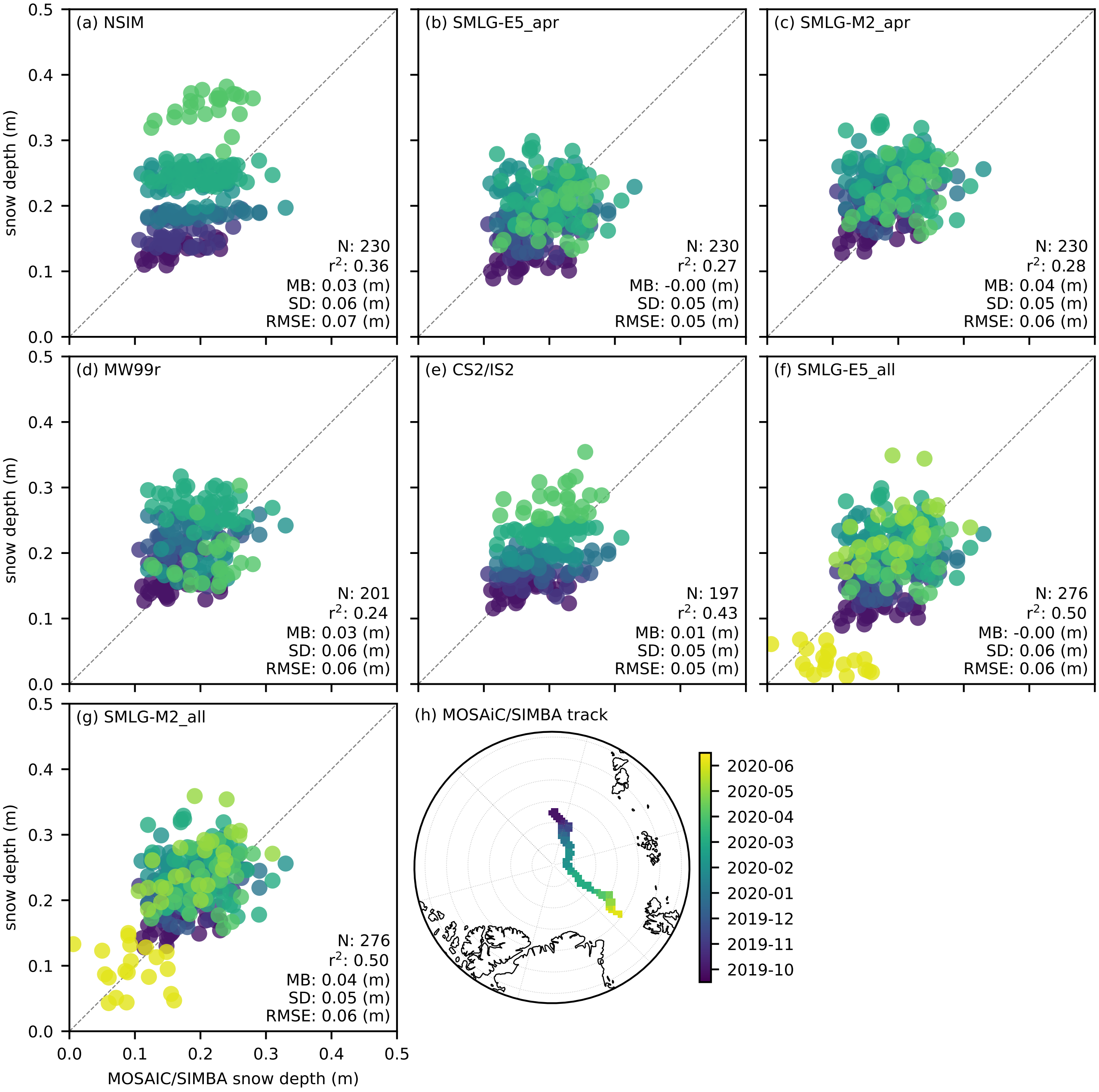

Fig. S9. Comparisons of October 2019 to June 2020 MOSAiC/SIMBA buoy snow depth measurements (binned to a 25 km North Polar Stereographic grid) and coincident monthly mean coarsened snow depths (sub-sampled by ICESat-2 and redistributed before gridding) from (a) NESOSIM v1.1 snow loading (NSIM) through to April 2020, (b) ERA5 forced SnowModel-LG (SMLG-E5) snow loading through to April 2020, (c) MERRA-2 forced SMLG snow loading through to April 2020, (d) modified Warren 99 snow loading (MW99) through April 2020, (e) IS2/CS2 dual altimetry fusion snow depths, (f) SMLG-E5 snow loading through to June 2020, (g) SMLG-M2 snow loading through to June 2020. Panel (h) shows the MOSAiC/SIMBA track color-coded by date, which are used in the colors across all scatter plots.
